# Deciphering ERR family genes as prognostic and immunological biomarkers through pan-cancer analysis with validation in gallbladder cancer

**DOI:** 10.3389/fonc.2025.1525635

**Published:** 2025-04-28

**Authors:** Wanwan Gong, Sijia Wen, Yu Chen, Fan Wu, Mengmeng Yang, Ping Sun, Xingmei Guo, Meiqin Li, Daozhen Chen, Hui Zhao, Lei Wang

**Affiliations:** ^1^ Department of Hepatopancreatobiliary Surgery, Jiangnan University Medical Center, Wuxi, China; ^2^ Wuxi School of Medicine, Jiangnan University, Wuxi, China; ^3^ Research Institute for Reproductive Health and Genetic Diseases, Wuxi Maternal and Child Health Hospital, Wuxi School of Medicine, Jiangnan University, Wuxi, China; ^4^ Jiangsu Provincial Key Laboratory on Parasite and Vector Control Technology, Jiangsu Institute of Parasitic Diseases, Wuxi, China; ^5^ Department of Pathology, Jiangnan University Medical Center, Wuxi, China; ^6^ Research Institute for Reproductive Health and Genetic Diseases, The Affiliated Wuxi Maternity and Child Health Care Hospital of Nanjing Medical University, Wuxi, China; ^7^ Department of Hepatopancreatobiliary Surgery, The Affiliated Wuxi No.2 People’s Hospital of Nanjing Medical University, Wuxi, China

**Keywords:** ERRs, pan-cancer, immune invasion, PD-L1, ESRRG, gallbladder cancer

## Abstract

**Background:**

The estrogen-related receptor family genes (ERRs), including ESRRA, ESRRB, and ESRRG, have been implicated in a few tumors, exhibiting distinct roles through diverse mechanisms. The purpose of our research is to explore the commonalities and underlying mechanism of ERRs in malignancies from a pan-cancer perspective and to validate the role and mechanisms of ESRRG in gallbladder cancer (GBC).

**Methods:**

We leveraged public databases such as TCGA and GTEx to systematically investigate the potential functions of ERRs in malignancies. ESRRG expression was analyzed through immunohistochemical staining in gallbladder cancer and cholecystitis tissues. For functional validation, ESRRG was knocked down in GBC cell lines, followed by CCK-8, colony formation, scratch wound healing, Transwell migration, and invasion assays. Western blot, qPCR, and immunofluorescence were performed to evaluate the relationship between ESRRG, PD-L1, and CD8^+^ T cells.

**Results:**

Compared to adjacent normal tissues, ESRRA is overexpressed in most tumors, ESRRB is generally underexpressed, and ESRRG exhibits significant expression alterations across various tumors. All three ERRs demonstrate significant prognostic value across different cancers. Notably, the strong associations of ERRs with key immunological features—stromal scores, immune cell infiltration, microsatellite instability (MSI), and tumor mutational burden (TMB)—suggest their involvement in immune evasion and their potential utility in guiding immunotherapy strategies. All three ERRs display a positive correlation with advanced tumor stages in cholangiocarcinoma (CHOL). Specifically, in CHOL, ESRRG expression is closely associated with lymphatic metastasis, poorer overall survival, reduced immune infiltration, elevated PD-L1 expression, epithelial-mesenchymal transition (EMT), and DNA damage response. In GBC tissues, we subsequently confirmed that ESRRG expression positively correlates with pathological staging and PD-L1 expression, while negatively correlating with prognosis and CD8^+^ T cell infiltration. Knockdown of ESRRG in gallbladder cancer cells results in decreased proliferation, migration, and invasion. Moreover, the expression of PD-L1, MSH2, BRCA1, MMP2, and VIMENTIN decreased with ESRRG knockdown.

**Conclusion:**

Our pan-cancer analysis reveals ERRs as critical regulators of tumor immunity and progression, with ESRRG emerging as a key oncogenic driver in GBC. The mechanistic link between ESRRG and PD-L1/EMT suggests its potential as a therapeutic target to enhance immunotherapy efficacy. These findings underscore the need for tissue-specific targeting strategies for ERR family members in precision oncology.

## 1 Introduction

Recent pan-cancer studies have significantly enriched researchers’ understanding of how the tumor immune microenvironment (TIME) influences tumorigenesis, progression, and therapeutic outcomes (1). The TIME encompasses various immune cells and signaling molecules that are critical to tumor development and substantially influence treatment outcomes (2). The existence of similar TIME patterns across multiple cancer types suggests that an immunotherapy strategy effective for one type of cancer might be applicable to others, albeit with varying degrees of success. The presence of cytotoxic T cells, natural killer cells, myeloid-derived suppressor cells, and regulatory T cells within the TIME can serve as predictive markers for a patient’s responsiveness to immunotherapy. It has been demonstrated that immune checkpoint inhibitors (ICIs) improve treatment outcomes in a variety of cancers (3). Increased levels of immune checkpoint molecules like programmed cell death ligand 1 (PD-L1) are related to enhanced sensitivity to ICI therapy in certain malignancies (4, 5).

The estrogen-related receptor family genes (ERRs), comprising ESRRA (ERRα), ESRRB (ERRβ), and ESRRG (ERRγ), are structurally analogous to the estrogen receptor gene family but function as nuclear orphan receptors due to their lack of endogenous ligands (6). ERRs are implicated in cancer progression through their regulation of metabolism, transcription, and related signaling pathways, highlighting their potential as therapeutic targets. Among the ERRs, ESRRA is the most extensively studied member, known to participate in mitochondrial function and the transcriptional regulation of downstream targets. There is a negative association between ESRRA and the prognosis in breast and ovarian malignancies, as it facilitates tumor growth and metastasis by modulating metabolic pathways and energy homeostasis (7). Recent evidence also suggests that ESRRA can modulate TIME, enhancing angiogenesis and immune evasion. Inhibiting ESRRA could not only induce cytokine activation, thus promoting the polarization of proinflammatory macrophages, but also stimulate antigen presentation, thus recruiting CD8^+^ T cells into TIME (8). Our previous study demonstrated that ESRRA enhances gallbladder cancer (GBC) proliferation via promoting Nectin-4 transcription, further supporting its role in tumor progression (9). ESRRB, though less studied, has shown tumor-suppressive properties in some cancers. For example, ESRRB expression has been linked to suppress growth of prostate cancer cells via p21 induction (10). Additionally, it has been found that ESRRB suppresses the viability and migration of malignant breast cells by upregulating p21 and E-cadherin (11). ESRRG plays a dual role in cancer, acting as an oncogene in some cancers while exhibiting tumor-suppressive functions in others. In hepatocellular carcinoma, high ESRRG expression correlates with increased tumor aggressiveness and poor clinical outcomes (12). An orally inverse agonist of ESRRG promotes ferroptosis in sorafenib-resistant hepatocellular carcinoma (13). Conversely, in breast cancer, it has been demonstrated that ESRRG suppresses cell proliferation and induces differentiation. Recent studies have also focused on the function of ERRs in TIME. ERRs have been shown to influence immune cell infiltration and effect within tumors. ESRRA has been found to enhance the infiltration level of neutrophil cells while decreasing that of CD4^+^ naïve T cells, NK cells, and cancer-associated fibroblasts (14). In cancers that are resistant to immunotherapy, ESRRA is abundantly expressed. Through cytokine induction and antigen-presentation stimulation, ESRRA inhibition induces tumor cytotoxicity and attracts CD8^+^ T lymphocytes to the tumor (8). This modulation of the immune microenvironment by ERRs suggests that targeting these receptors could enhance the effectiveness of immunotherapies. Advances in the regulation of ERRs have opened new therapeutic strategies. The integration of ERRs modulation with existing immunotherapies could enhance treatment efficacy and overcome resistance mechanisms.

While the roles and mechanisms of ERRs in a few malignancies have been preliminary reported, no study has comprehensively examined the functions of them from a pan-cancer view. In our research, we leverage public databases such as The Cancer Genome Atlas (TCGA) and the Genotype-Tissue Expression (GTEx) project and utilize platforms like Sangerbox and Xenashiny, along with the R programming language, to perform a pan-cancer analysis of ERRs for uncovering their impact on cancer development and prognosis. Additionally, to delve deeper into the mechanisms by which the ERRs influence cancer, we analyzed their associations with key immunological features such as cytokines, immune checkpoint genes, stromal scores, immune cell infiltration, tumor mutation burden (TMB), and microsatellite instability (MSI). The purpose of this investigation is to elucidate the influence of the ERRs on TIME, exploring their potential for combined immunotherapy applications. Lastly, guided by the pan-cancer analysis, we validated the impact of ESRRG on proliferation, migration, invasion, and PD-L1-mediated immune evasion in gallbladder cancer. By knocking down ESRRG, we investigated whether those signaling pathways and key factors of the Protein-Protein Interaction (PPI) network identified by the pan-cancer analysis mediated the related biological functions of ESRRG.

## 2 Results

### 2.1 Expression of ERR family genes in pan-cancer

**Figure 1:**
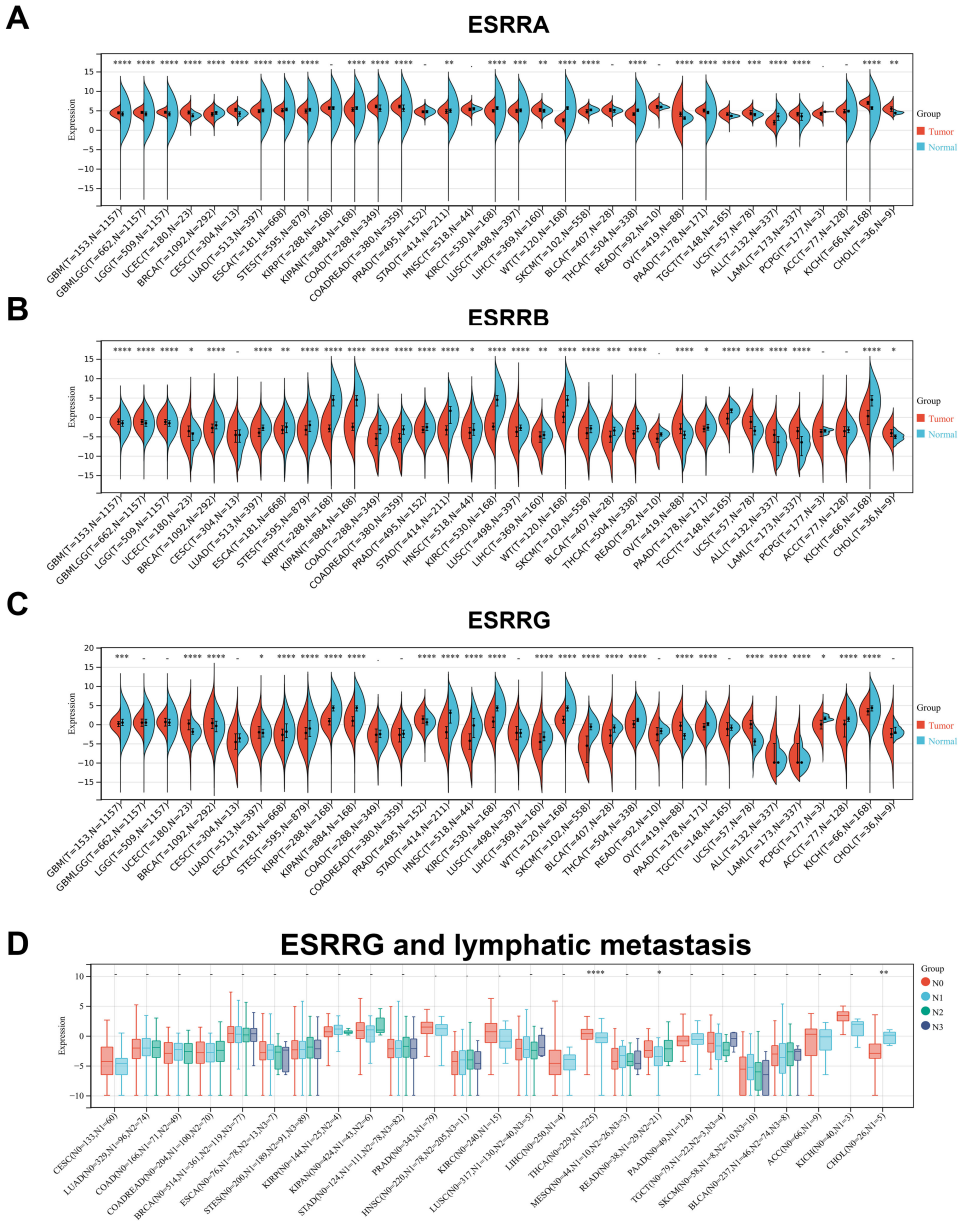
The expression levels of ERRs in pan-cancer. **(A-C)** The expression of ESRRA **(A)**, ESRRB **(B)** and ESRRG **(C)** in tumors tissues and corresponding nontumor tissues was analyzed using TCGA and GTEx databases. **(D)** The association of ESRRG with lymph node metastasis in pan-cancer. *p < 0.05; **p < 0.01; ***p < 0.001; ****p < 0.0001; p < 0.05 was considered statistically significant.

ERRs expression in 34 kinds of tumors was searched in TCGA and GTEx datasets. The expression in cancerous tissues differed significantly from those in adjacent normal tissues. ESRRA expression remained high in Glioblastoma (GBM), lower grade glioma and glioblastoma (GBMLGG), Lower Grade Glioma (LGG), Endometrioid Cancer (UCEC), Cervical Cancer (CESC), Colon Cancer (COAD), Colon and Rectal Cancer (COADREAD), Liver Cancer (LIHC), Bladder Cancer (BLCA), Ovarian Cancer (OV), Pancreatic Cancer (PAAD), Testicular Cancer (TGCT), Uterine Carcinosarcoma (UCS), Acute Myeloid Leukemia (LAML), Kidney Chromophobe (KICH), and CHOL, and low in Breast Cancer (BRCA), Lung Adenocarcinoma (LUAD), Esophageal Cancer (ESCA), Stomach and Esophageal carcinoma (STES), Pan-kidney cohort (KIPAN), Stomach Cancer (STAD), Kidney Clear Cell Carcinoma (KIRC), Lung Squamous Cell Carcinoma (LUSC), Wilms tumor (WT), Melanoma (SKCM), Thyroid Cancer (THCA), and Acute lymphoblastic leukemia (ALL) (**Figure 1A**). ESRRB exhibited high expression levels in GBM, GBMLGG, LGG, UCEC, OV, UCS, ALL, LAML, and CHOL, but was poorly expressed in BRCA, LUAD, ESCA, STES, KIRP, KIPAN, COAD, COADREAD, PRAD, STAD, Head and Neck Cancer (HNSC), KIRC, LUSC, LIHC, WT, SKCM, BLCA, THCA, Rectal Cancer (READ), PAAD, TGCT, and KICH (**Figure 1B**). ESRRG was upregulated in UCEC, BRCA, LUAD, Prostate Cancer (PRAD), OV, UCS, ALL, LAML, and downregulated in GBM, ESCA, STES, Kidney Papillary Cell Carcinoma (KIRP), KIPAN, STAD, HNSC, KIRC, LIHC, WT, SKCM, BLCA, THCA, PAAD, Pheochromocytoma & Paraganglioma (PCPG), Adrenocortical Cancer (ACC), and KICH (**Figure 1C**).

### 2.2 Correlation of ERRs with clinical and pathological stage

Following further analysis of ERR family gene expressions at different pathological stages, significant differences were observed in ESCA, KIPAN, THYM, and THCA for ESRRA (**Supplementary Figure S1A**), LUAD, KIRC, THCA, and READ tumors for ESRRB (**Supplementary Figure S1B**), and KIPAN, KIRC, Thymoma (THYM), THCA, TGCT, and KICH for ESRRG (**Supplementary Figure S1C**). It is noteworthy that, despite the lack of statistical significance for ESRRA in TGCT, CHOL, and Large B-cell Lymphoma (DLBC), a general tendency suggesting the high expression of ESRRA in advanced tumors was obvious, suggesting that ESRRA may have prognostic value in these tumor types. So is that of ESRRB in THYM, LIHC, TGCT, UCS, and CHOL, and ESRRG in COAD and CHOL. Importantly, all of the three subfamily genes showed a trend toward positive correlation with advanced tumors in CHOL. Due to the limited number of cases (n = 35, Stage I = 19, Stage II = 9, Stage IV = 7), no statistical significance was obtained. According to stratification analysis, ESRRG expression was statistically elevated in CHOL with lymphatic metastasis (**Figure 1D**), suggesting the metastasis-promoting role of ESRRG.

### 2.3 Survival analysis and ROC curves

Overall survival (OS) and progression-free survival (PFS) are two domains of the most effective prognostic indicators. Cox regression analysis indicates that ERRs influence the prognosis of multiple tumors. In concrete terms, high expression of ESRRA was suggestive of lower PFS in BRCA, PRAD, PCPG, and CHOL (**Supplementary Figure S2A**), as well as lower OS in THYM, LAML, and ALL-R (**Supplementary Figure S2B**). Poorer PFS in GBMLGG, LGG, and THYM (**Supplementary Figure S2C**) and poorer OS in GBMLGG, LGG, COAD, COADREAD, THYM, THCA, and ACC (**Supplementary Figure S2D**) were linked to high expression of ESRRB. ESRRG expression was negatively associated with PFS in carcinoma categories including STES, STAD, and ACC (**Supplementary Figure S2E**). High ESRRG expression tended to correlate with lower OS in patients with THCA, ACC, STAD, THYM, LGG, and CHOL (**Supplementary Figure S2F**). Notably, in THYM, all these three genes were risk factors for OS. Meanwhile, in a variety of cancer types, increased expression of these three genes has been favorably correlated with improved PFS and OS (**Supplementary Figures S2A-F**). Represented by the relationship between ESRRA and PFS in KIPAN, KIRC, KIRP, and GBMLGG. In KIPAN, all these three genes were protective factors for OS. Based on the above results, ERR family genes may play opposite effects on prognosis in various cancer types. The associations between ERR genes and OS or PFS were additionally explored through Kaplan-Meier survival analysis. The observed trends closely mirrored those identified in the multivariate Cox regression analysis (**Supplementary Figures S3, S4**). Notably, in CHOL, ACC, PRAD, STAD, and THCA, either PFS or OS was associated with the expression of more than one ERR family gene (**Supplementary Figure S3**).

Subsequently, survival prediction was made by ROC curves of 42 cancer types. The findings suggested that ESRRA was highly accurate (AUC>0.7) in predicting the survival of Mesothelioma (MESO) and PCPG (**Supplementary Figures S5A, B**), and was highly accurate (AUC>0.7) in predicting the progression of CHOL (**Supplementary Figure S5C**). Moreover, models with accuracy (AUC>0.65) for survival prediction were ESRRA for KIPAN, KIRP, and PRAD (**Supplementary Figures S5D-F**), and ESRRB for THCA and THYM (**Supplementary Figures S5G, H**). Models with accuracy (AUC>0.65) for progression prediction were ESRRG for ACC and KIRC (**Supplementary Figures S5I, J**). It is evident that the prognosis of many cancers is significantly influenced by the ERR gene family. However, due to the heterogeneous impact of ERR genes on prognosis, it is necessary to determine their role in various cancers through more extensive specimen sequencing and experimental verification.

### 2.4 ERR family genes are tightly related to immune checkpoints and immune infiltration

In OV, ESRRA exhibited predominantly positive correlations with immune infiltrating genes, whereas negative correlations were prevalent in KIPAN and THCA. (**Supplementary Figure S6A**). ESRRB displayed a prevailing trend of positive correlations with immune infiltrating genes in LGG, GBMLGG, and LUAD, whereas negative correlations predominated in TCTG (**Supplementary Figure S6B**). ESRRG was predominantly negatively correlated with ACC and BLCA (**Supplementary Figure S6C**). Meanwhile, in most cancer types, a noteworthy positive connection was observed between ESRRA and TAP1 (belonging to MHC) and TNFRSF14 (**Supplementary Figure S6A**). A close positive correlation between ESRRG and CD160 was also found in predominant cancer types (**Supplementary Figure S6C**). TNFRSF14 and CD160 served as immune checkpoint stimulators and immune checkpoint inhibitors, respectively, indicating a close regulatory relationship between ERR genes and immune regulation.

Higher stromal score, immune score, and estimate score indicated lower purity of the tumor and better prognosis. In most kinds of cancer, ESRRA and ESRRG had a negative correlation with stromal score, while ESRRB had a positive correlation with stromal score (**Supplementary Table S1**). An R value greater than 0.4 was defined as a strong correlation. ESRRA was strongly negatively correlated with ESCA, STES, KIPAN, KIRC and PAAD and positively correlated LAML (**Supplementary Figures S6D, S7A, B**). ESRRB has strong positive correlation with COAD and COADREAD (**Supplementary Figure S6E**). No strong correlation was found between ESRRG and stromal score (**Supplementary Figure S6F**). All ERR genes except ESRRB exhibited a negative correlation with immune score. To be specific, ESRRA was highly negatively associated with KIPAN (**Supplementary Figure S6G**). ESRRB was highly positively associated with LGG (**Supplementary Figure S6H**), while ESRRG was highly negatively associated with GBMLGG, LGG, THCA, and ACC (**Supplementary Figures S6I, S7C**). Among all ERR genes, but not ESRRB, expression was mostly negatively correlated with the estimate score. In particular, ESRRA was closely negatively related to estimate score in STES, KIPAN, STAD and KIRC (**Supplementary Figures S6J, S7D**). ESRRB was closely positively related to the estimate score in LGG, COAD, and COADREAD (**Supplementary Figure S6K**), while ESRRG was closely negatively related to LGG and ACC (**Supplementary Figure S6L**). The above results hinted that ESRRA and ESRRG usually served as oncogenes, while ESRRB may be a cancer suppressor gene in most cancers.

### 2.5 Correlation of ERR family genes with immune infiltrating cells

**Figure 2:**
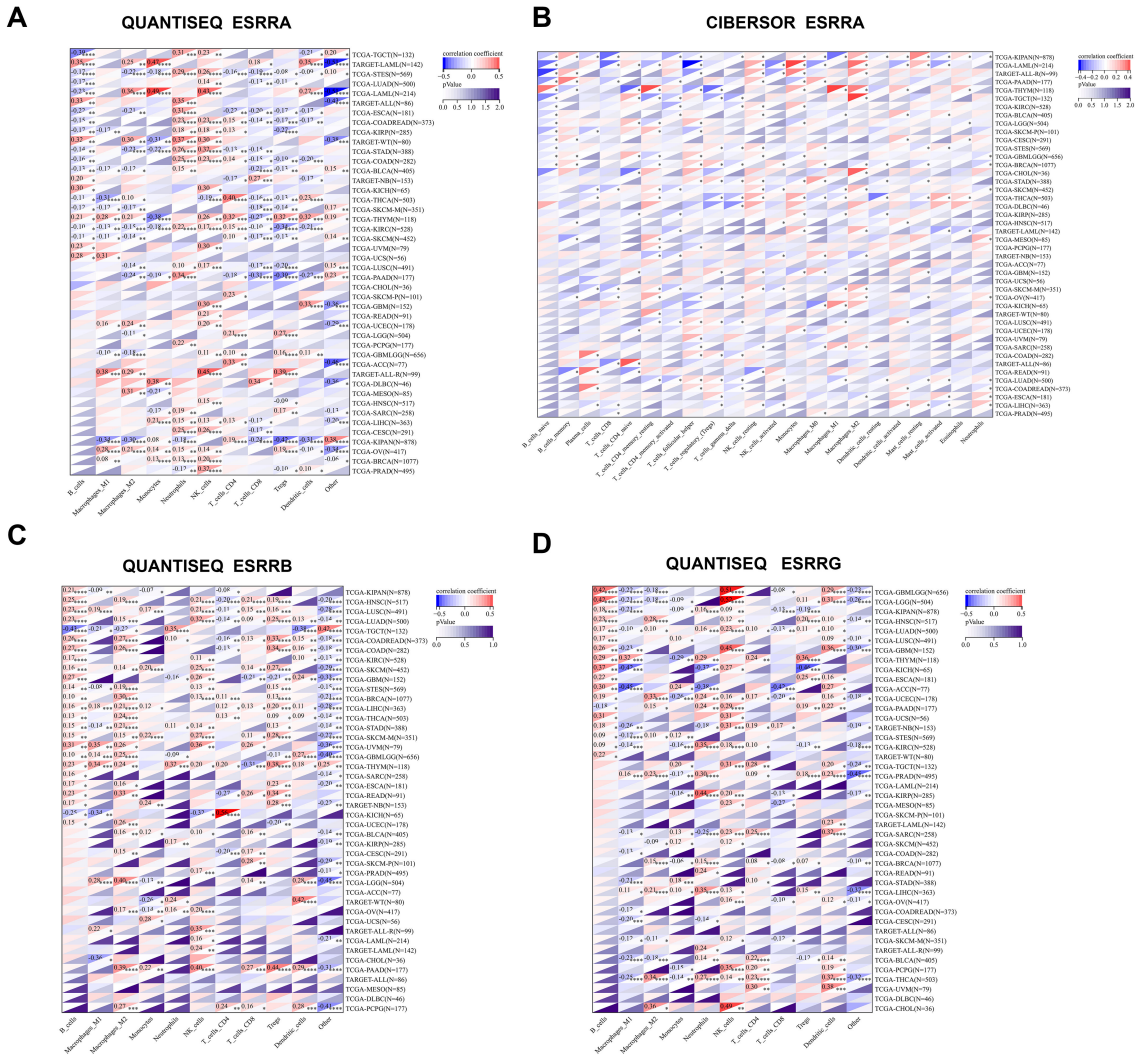
Expression of ERRs is related to the level of immune infiltrating cells. **(A-B)** Correlation of ESRRA with the level of immune infiltrating cells using QUANTISEQ **(A)** and CIBERSORT **(B)** algorithms. **(C-D)** Correlation of ESRRB **(C)** and ESRRG **(D)** with the level of immune infiltrating cells using QUANTISEQ. *p < 0.05; **p < 0.01; ***p < 0.001; ****p < 0.0001; p < 0.05 was considered statistically significant.

We further incorporated three well-established algorithms—QUANTISEQ, CIBERSORT, and TIMER—to evaluate cross-tumor immune scoring. This allowed us to evaluate the relationship of ERRs expressions with immune cell levels, expanding upon our investigation of immune infiltration. By combining the results from these algorithms, we found that in most cancers, ESRRA strongly contributed to the infiltration of natural killer cells and neutrophils, and weakened the infiltration of CD8^+^ T cells (**Figures 2A, B**; **Supplementary Figure S8A**). In LAML, ALL-R, THYM, and CHOL, CIBERSOR or QUANTISEQ revealed that ESRRA were highly associated with macrophages M2 (**Figures 2A, B**), indicating an immunosuppressive microenvironment. TIMER demonstrated that ESRRA promoted the infiltration of DC, neutrophils, CD4^+^T cells, and B cells in estrogen related cancers OV and BRCA (**Supplementary Figure S8A**). In predominant cancer types, QUANTISEQ revealed the close correlation of ESRRB with the infiltration level of B cells and macrophages M2 (**Figure 2C**), while CIBERSOR showed a substantial contribution of ESRRB to Treg infiltration in CHOL (**Supplementary Figure S8B**). TIMER demonstrated significant positive interaction between ESRRB and all six types of immune cells (**Supplementary Figure S8C**). In most cancers, QUANTISEQ showed that ESRRG strongly contributed to the infiltration of natural killer cells and B cells, and weakened the infiltration of macrophages M1 and CD8^+^ T cells, promoting the formation of an immunosuppressive microenvironment (**Figure 2D**). The QUANTISEQ algorithm further showed positive a correlation of ESRRG with macrophages M2 in CHOL, THCA, and UCEC et al, and with dendritic cells in Ocular melanomas (UVM), GBM, THCA, and Sarcoma (SARC) et al. (**Figure 2D**). Different from the QUANTISEQ algorithm, the CIBERSOR algorithm showed a weak correlation between ESRRG and natural killer cells (**Supplementary Figure S8D**), and the TIMER algorithm showed a strong positive correlation between ESRRG and CD8^+^ T cells in PRAD, THCA, KICH, and UCS (**Supplementary Figure S8E**), which hints at the necessity of delving into the role of immune cells in these tumors.

### 2.6 Prediction of sensitivity of anti-PD-1/PD-L1 antibody by ERR family genes

In order to assess the sensitivity of immune checkpoint inhibitors, such as anti-PD-1/PD-L1, we analyzed the correlation between ERRs and MSI and TMB at the pan-cancer level. ESRRA mRNA expression was most tightly correlated with TMB scores in CHOL, PRAD, SKCM, STAD, and THYM (**Supplementary Figure S9A**), while ESRRA expression in patients with UCEC, LGG, and LUAD was positively related to MSI (**Supplementary Figure S9B**). ESRRB was negatively linked to TMB scores in KIRC, LUSC, MESO, and STAD (**Supplementary Figure S9C**), meanwhile a negative association between ESRRB and MSI was presented in BRCA and LUAD (**Supplementary Figure S9D**). ESRRG displayed a prominent negative correlation with TMB in ACC, COAD, KIRC, LUAD, LUSC, OV, STAD, and THCA (**Supplementary Figure S9E**), and a negative correlation with MSI in COAD and STAD (**Supplementary Figure S9F**). The positive correlation of ESRRB with TMB score and the negative correlation of ESRRG with TMB score in CHOL were obvious, although no statistical significance was reached due to limited cases (**Supplementary Figures S9C, E**). The stemness score of tumor cells is associated with malignant proliferation and drug resistance. The spearman correlation between the stemness score of various cancer types and ERR family gene expressions was evaluated. Predominant cancer types exhibited a positive correlation between ESRRA and stemness scores (**Supplementary Figure S9G**), while a negative correlation between stemness scores and ESRRB (**Supplementary Figure S9H**) as well as ESRRG (**Supplementary Figure S9I**) was established.

### 2.7 ERRG could be an oncogene in CHOL represented by gallbladder cancer

**Figure 3:**
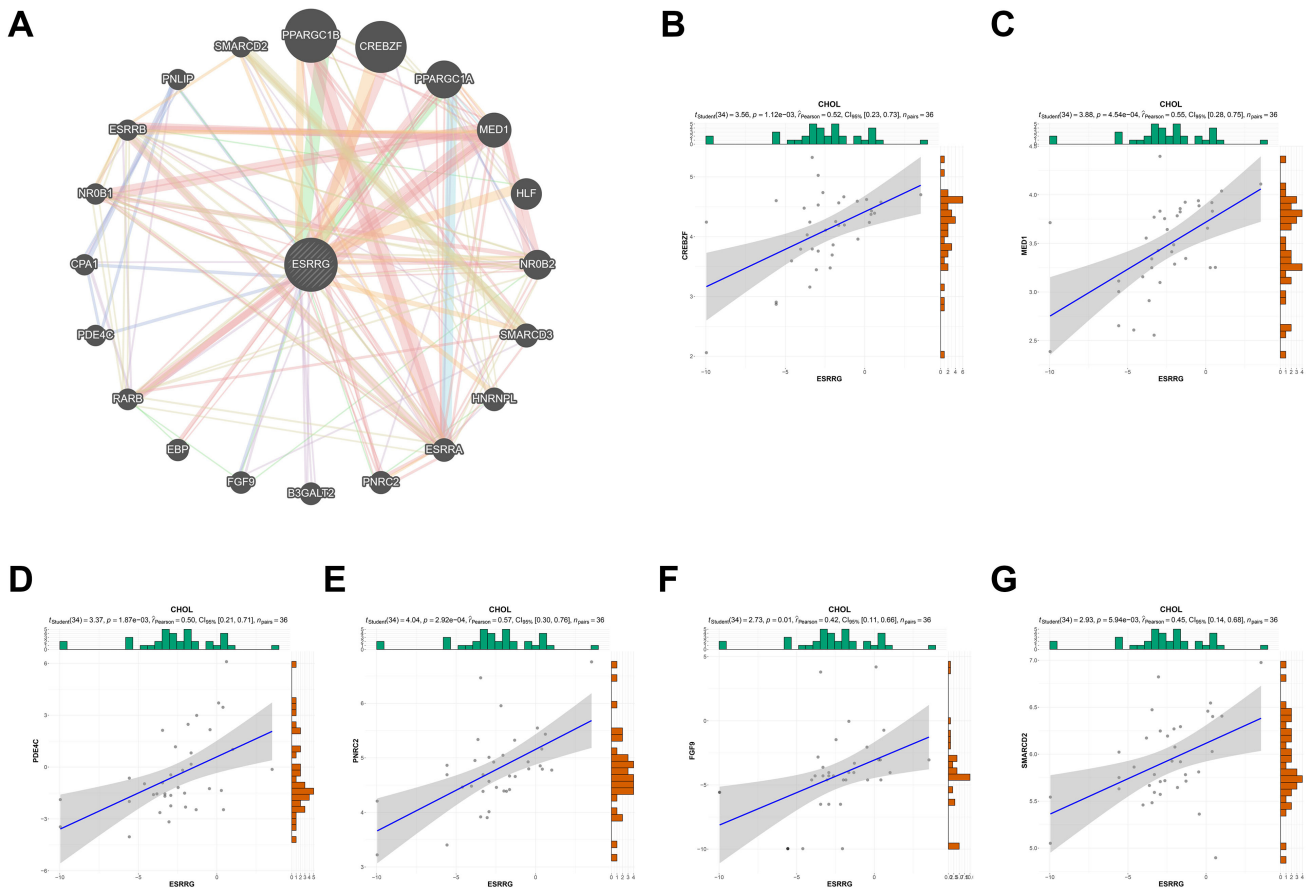
PPI network of ESRRG. **(A)** The potential interaction molecular network of ESRRG was created by PPI. **(B-G)** Validation of the correlation between ESRRG expression and related genes of PPI network in CHOL.

Among the 44 cancer types in TCGA TARGET GTEx datasets, CHOL had the smallest number of cases analyzed. Although there was a trend for correlation between ERR genes and prognosis, stage, and immunoinfiltration scores, there was no statistical significance due to limited cases. Based on the available data, bioinformatics analysis alone could not fully determine the role of ERR family in CHOL. Therefore, cytological and histological verification were urged. We have confirmed the role of ESRRA in promoting CHOL in preliminary experiments (9, 15), while the role of ESRRB and ESRRG in CHOL has not been confirmed experimentally. Notably, the above results confirmed that, in CHOL, ESRRG expression was higher in patients with advanced stage and lymphatic metastasis. Moreover, patients with higher ESRRG expression exhibited poorer overall survival and lower immune infiltration. Based on the results of Gene Set Variation Analysis (GSVA) (16, 17), the association of ESRRG with pathway scores was explored. The result showed that ESRRG was closely related to the activation of DNA damage response, hormone AR, hormone ER, PI3K/AKT pathway, Ras/MAPK pathway, RTK pathway, and Tsc/mTOR pathway (**Supplementary Figure S10A**). Some key regulators of these pathways, such as MSH2, MLH1, BRCA1, VIMENTIN, MMP2, and AKT1, were identified to be significantly related to ESRRG expression by the XenaShiny TCGA Association Analysis module for single cancer (**Supplementary Figures S10B-N, S11**). In eight cancer types, including CHOL, there was a positive correlation found between the expression of PD-L1 and ESRRG (**Supplementary Figure S12**). The PPI network identified that ESRRG was significantly related to 20 genes, including PPARGC1A, PPARGC1B, MED1, CREBCF and so on (**Figure 3A**), 6 of which were confirmed to be positively correlated with ESRRG expression by the XenaShiny TCGA Association Analysis module for single cancer (**Figures 3B-G**). Therefore, we next conduct some experiments to explore the function of ESRRG in CHOL, represented by gallbladder cancer, and the possibility of the above mechanisms.

### 2.8 ESRRG expression in IHC was linked to advanced stage and poor survival

**Figure 4:**
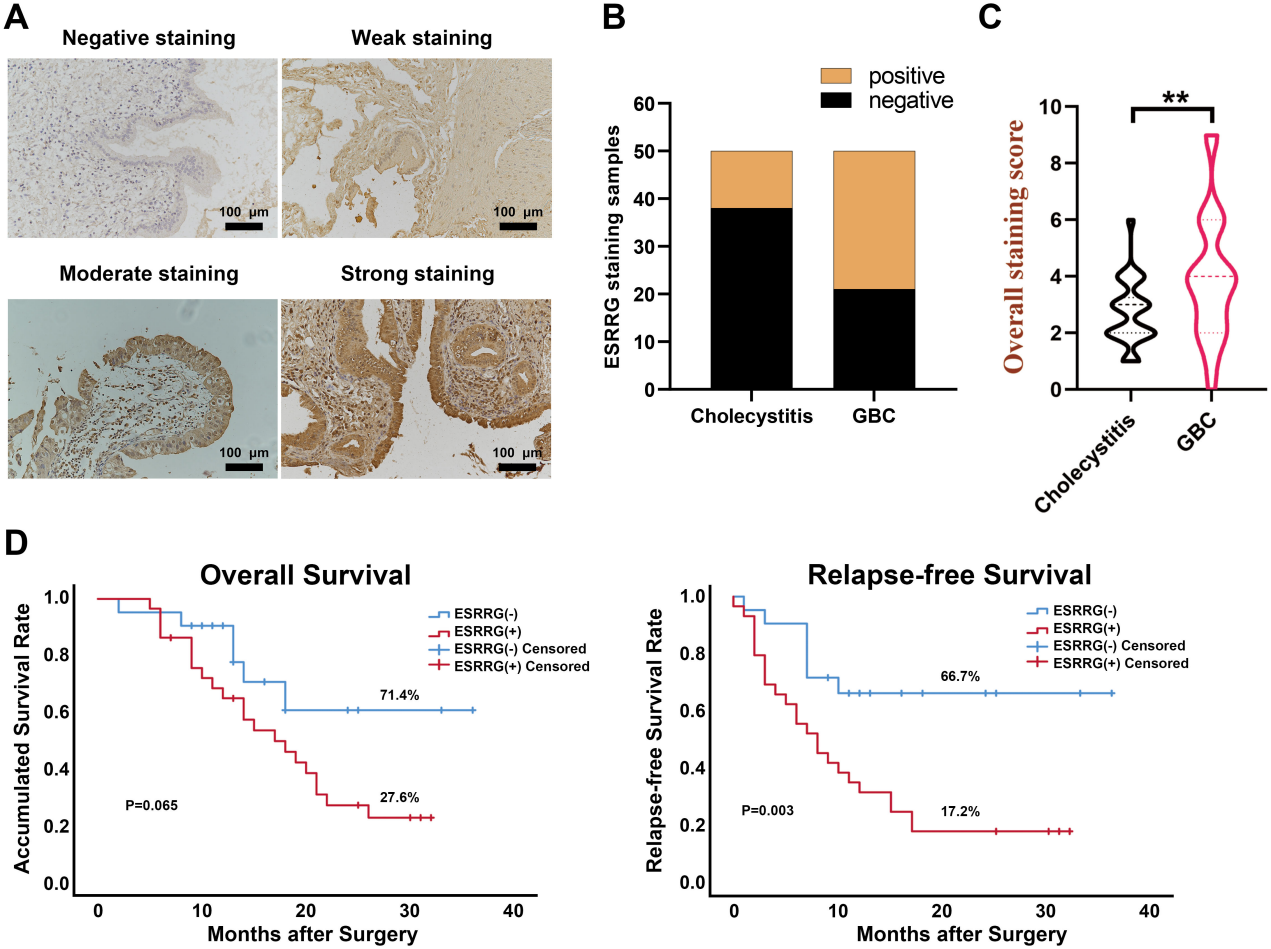
ESRRG is highly expressed in GBC tissues compared with cholecystitis tissues. **(A)** Representative immunohistochemical staining images of ESRRG with staining intensity of “negative”, “weak”, “moderate” and “strong”. (original magnification, ×200). **(B)** The expression of ESRRG is higher in GBC tissues compared to cholecystitis tissues. The final immunereactivity score was calculated by multiplying the intensity and percentage scores. Scores from 0 to 3 were considered “negative”, while scores from 4 to 12 were deemed “positive”. **(C)** IHC score of ESRRG in GBC and cholecystitis tissues. **(D)** ESRRG expression is correlated with patients’ OS and RFS. The symbol "**" denotes statistical significance at p < 0.01.

In our prior pan-cancer analysis, we hypothesized that ESRRG had a positive association with lymph node metastasis in cholangiocarcinoma (CHOL). Although ESRRG showed a tendency to positively correlate with advanced tumors in CHOL, the limited number of CHOL cases in the database prevented statistical significance. To investigate this potential correlation, we performed immunohistochemical analysis on ESRRG expression in 50 gallbladder cancer (GBC) samples and 50 cholecystitis samples. We then analyzed the correlation of ESRRG with clinicopathological staging and prognosis. Representative IHC pictures are presented in **Figure 4A**. Our study revealed that 58% (29/50) of GBC samples exhibited positive ESRRG expression, significantly higher than the positive rate (24%, 12/50) observed in cholecystitis samples (**Figure 4B**). Moreover, compared to cholecystitis, the expression level of ESRRG in GBC was much greater (**Figure 4C**). High ESRRG expression in GBC was significantly associated with advanced TNM stage, deeper invasion, and lymph node metastasis (**Table 1**). The analysis of Kaplan-Meier survival indicated that positive ESRRG expression correlates with poor PFS (**Figure 4D**, p < 0.01).Although ESRRG expression showed a negative correlation with poorer OS (**Figure 4D**), this association did not reach statistical significance (p = 0.065), suggesting that ESRRG may play a more critical role in tumor recurrence and metastasis. Furthermore, multivariate survival analysis revealed ESRRG to be an independent prognostic and recurrent indicator (**Tables 2**, **3**) in GBC.

**Table 1:** Correlations of ESRRG expression with clinicopathological features of GBC.

Clinicopathological features	Total cases	ESRRG expression level		p
	N	Negative N (%)	Positive N (%)	
Gender Male Female	22 28	8 (36.4) 13 (46.4)	14 (63.6) 15 (53.6)	0.474
Age (years) <60 ≥60	10 40	3 (30) 18 (45)	7 (70) 22 (55)	0.390
Histology WD+MD PD+UD	29 21	16 (55.2) 5 (23.8)	13 (44.8) 16 (76.2)	0.027*
Resection margin Negative Positive	44 6	20 (45.5) 1 (16.7)	24 (54.5) 5 (83.3)	0.380
Pathologic T stage Tis + T1 + T2 T3 + T4	35 15	19 (54.3) 2 (13.3)	16 (45.7) 13 (86.7)	0.007**
Lymph node metastasis Absent Present	33 17	17 (51.5) 4 (23.5)	16 (48.5) 13 (76.5)	0.058
Distant metastasis Absent Present	47 3	21 (44.7) 0 (0.0)	26 (55.3) 3 (100.0)	0.254
TNM state (AJCC) 0-II III–IV	27 23	17 (63.0) 4 (17.4)	10 (37.0) 19 (82.6)	0.001**

WD, well differentiated; MD, moderately differentiated; PD, poorly differentiated; UD, undifferentiated; *p < 0.05; **p < 0.01. p < 0.05 was considered statistically significant.

**Table 2:** Univariate and multivariate analysis of prognostic factors in GBC patients.

Variables	Unfavorable/ favorable	Univariate analysis		Multivariate analysis	
		HR (95% CI)	p	HR (95% CI)	p
Gender	Female/Male	1.22(0.569-2.619)	0.609		
Age	≥60/<60	2.015(0.689-5.893)	0.201		
Histology	PD+UD/WD+MD	2.799(1.291-6.068)	0.009**		
Resection margin	R1/R0	4.794(1.839-12.495)	0.001**	5.825(2.173-15.619)	0.000**
Pathologic T stage	T3+T4/Tis+T1+T2	1.257(0.572-2.765)	0.569		
Lymph node metastasis	Present/Absent	1.923(0.902-4.100)	0.090		
Distant metastasis	Present/Absent	5.574(1.600-19.425)	0.007**	7.798(2.148-28.308)	0.002**
TNM state (AJCC)	III-IV/0-II	2.168(0.972-4.834)	0.059		
ESRRG expression	Positive/Negative	2.276(0.915-5.660)	0.077		

WD, well differentiated; MD, moderately differentiated; PD, poorly differentiated; UD, undifferentiated; R0, negative resection margin; R1 positive resection margin; CI, confidence interval; HR, hazard ratio. *p < 0.05; **p < 0.01. p < 0.05 was considered statistically significant.

**Table 3:** Univariate and multivariate analysis of recurrent factors in GBC patients.

Variables	Unfavorable/ favorable	Univariate analysis		Multivariate analysis	
		HR (95% CI)	p	HR (95% CI)	p
Gender	Female/Male	1.014(0.499-2.060)	0.970		
Age	≥60/<60	1.543(0.588-4.048)	0.378		
Histology	PD+UD/WD+MD	3.163(1.531-6.533)	0.002**	2.429(1.117-5.284)	0.025
Resection margin	R1/R0	6.044(2.227-16.399)	0.000**	4.474(1.484-13.493)	0.008**
Pathologic T stage	T3+T4/Tis+T1+T2	1.785(0.864-3.690)	0.118		
Lymph node metastasis	Present/Absent	2.104(1.033-4.283)	0.040*		
Distant metastasis	Present/Absent	3.535(1.060-11.792)	0.040*		
TNM state (AJCC)	III-IV/0-II	2.357(1.142-4.865)	0.020*		
ESRRG expression	Positive/Negative	3.285(1.413-7.640)	0.006**	2.731(1.149-6.488)	0.023

WD, well differentiated; MD, moderately differentiated; PD, poorly differentiated; UD, undifferentiated; R0, negative resection margin; R1 positive resection margin; CI, confidence interval; HR, hazard ratio. *p < 0.05; **p < 0.01. p < 0.05 was considered statistically significant.

### 2.9 ESRRG knockdown inhibits the proliferation and migration in GBC cells

**Figure 5:**
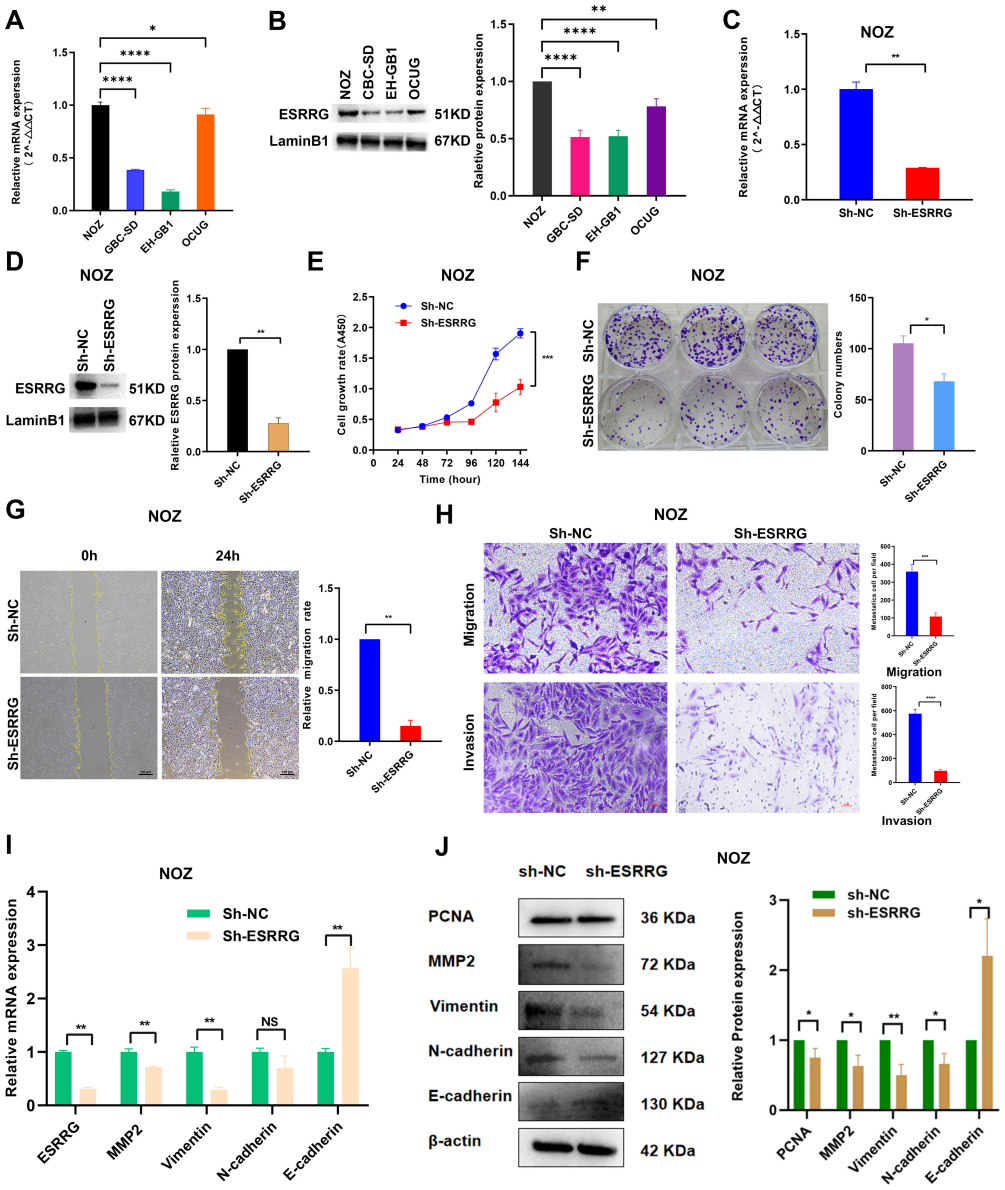
ESRRG promotes the malignant biological behavior of gallbladder cancer cells. **(A-B)** The expression of ESRRG in four GBC cell lines was assessed using Real-time PCR **(A)** and Western Blot **(B)**. **(C-D)** The knockdown effect of ESRRG in NOZ cells was determined using Real-time PCR **(C)** and Western Blot **(D)**. **(E-F)** CCK-8 **(E)** and colony formation assays **(F)** were performed to evaluate that the knockdown of ESRRG reduced the proliferation of NOZ cells. **(G-H)** Wound healing **(G)** and transwell assays **(H)** were conducted to demonstrate that ESRRG knockdown weakened the migration and invasion of NOZ cells. **(I-J)** qPCR **(I)** and Western blot **(J)** show that knockdown of ESRRG downregulated PCNA, MMP-2, VIMENTIN, and N-cadherin, while upregulating E-cadherin expression.

In order to further define the function of ESRRG in GBC, we performed subsequent cell-level experiments. Initially, we quantified the levels of ESRRG expression in four GBC cell lines, with the NOZ cell line exhibiting the highest expression (**Figures 5A, B**). Subsequently, we utilized RFect reagent to transfect shRNA into NOZ cells to knock down ESRRG expression (**Figures 5C, D**). CCK-8 and colony formation assays were conducted for assessing the impact of ESRRG on cell proliferation. In contrast to negative controls, the knockdown of ESRRG dramatically decreased the vitality and capacity of NOZ cells to form colonies (**Figures 5E, F**, p < 0.01), indicating that silencing ESRRG markedly inhibits the proliferative and clonogenic capacities of GBC cells. To investigate the effects of ESRRG on cell migration and invasion, we conducted wound healing and Transwell assays. The results revealed that sh-ESRRG significantly impaired the wound healing capability of NOZ cells relative to the negative controls (**Figure 5G**, p < 0.01). In addition, ESRRG silencing may considerably limit NOZ cell migration and invasion, as seen by the significantly lower number of migrating and invading cells in the sh-ESRRG group (**Figure 5H**, p < 0.01). Furthermore, we analyzed the relationship between ESRRG and epithelial-mesenchymal transition (EMT) markers and PCNA via qPCR (**Figure 5I**) and Western blot (**Figure 5J**) assays. The results demonstrated that knockdown of ESRRG downregulated PCNA, MMP-2, VIMENTIN, and N-cadherin, while upregulating E-cadherin expression, suggesting that ESRRG may promote tumor migration and invasion by modulating EMT. In summary, these results suggest the importance of ESRRG in facilitating the growth, migration, and invasion of gallbladder cancer cells. And it also implies that ESRRG may be an oncogenic component in the pathogenesis of gallbladder cancer.

### 2.10 ESRRG promotes the formation of an immunosuppressive microenvironment in GBC

**Figure 6:**
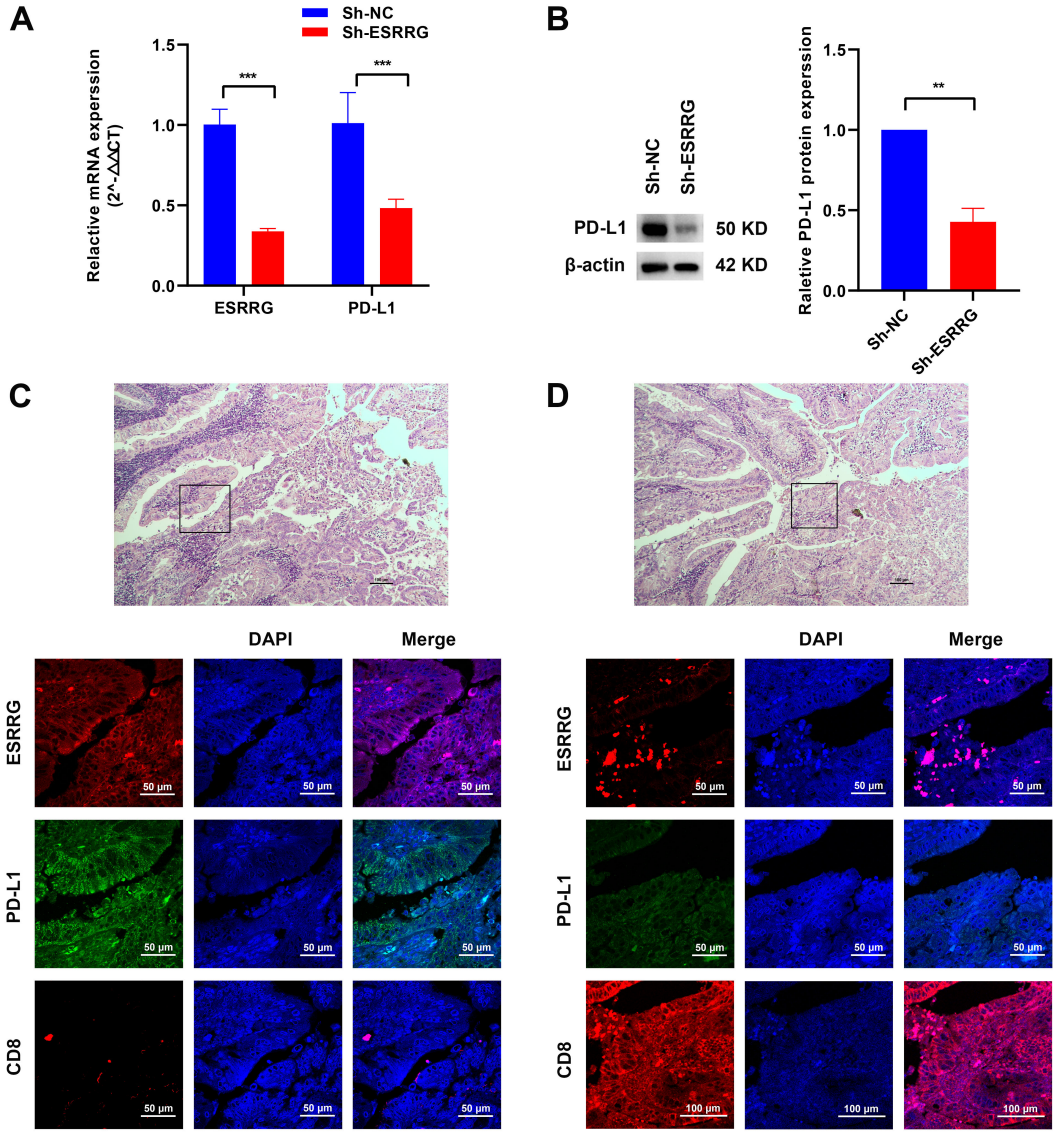
ESRRG is positively correlated with PD-L1. **(A-B)** The positive correlation between ESRRG and PD-L1 in GBC cells was investigated using Real-time PCR **(A)** and Western Blot **(B)**. **(C-D)** The expressions of ESRRG, PD-L1 and CD8 were detected by immunofluorescence assay in GBC tissues. **p < 0.01; ***p < 0.001; p < 0.05 was considered statistically significant.

Based on previous bioinformatics analysis, we hypothesized that ESRRG might contribute to the formation of an immunosuppressive microenvironment in various tumors. Gene correlation analysis demonstrated a positive association between ESRRG and PD-L1 expression, although statistical significance was not achieved for the limited number of samples (**Supplementary Figure S12H**). To further investigate this potential relationship in GBC, we carried out both *in vivo* and *in vitro* investigations. We found that ESRRG knockdown in NOZ cells significantly reduced PD-L1 expression (**Figures 6A, B**). Immunofluorescence analysis of tumor tissues showed that regions with high ESRRG expression had elevated PD-L1 level and decreased CD8^+^ lymphocyte infiltration (**Figure 6C**), while regions with low ESRRG expression had decreased PD-L1 level and upregulated CD8^+^ lymphocyte infiltration (**Figure 6D**). The above results suggested that ESRRG may promote an immunosuppressive microenvironment in gallbladder cancer.

### 2.11 Regulation of gene expression and functional pathways by ESRRG

**Figure 7:**
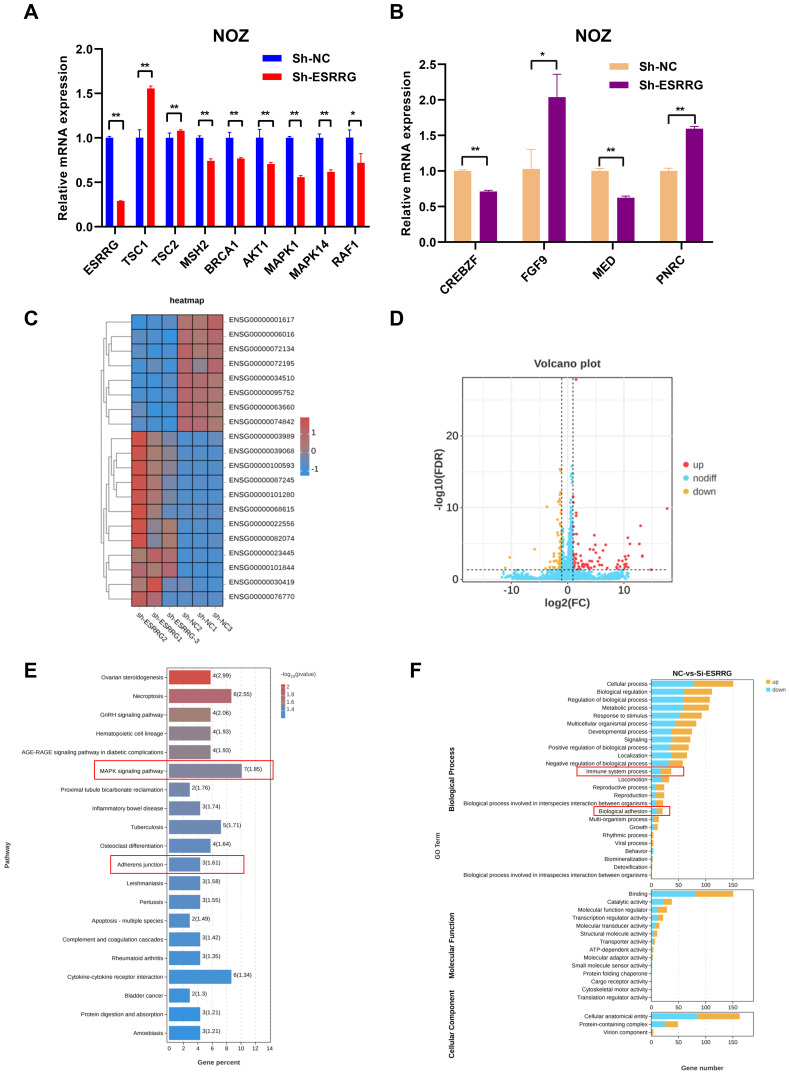
Validation of the correlation between ESRRG and key factors from GSVA and PPI analysis. **(A)** Alterations in related signaling regulators from GSVA analysis at the mRNA level following ESRRG knockdow. **(B)** Alterations in related genes from PPI network at the mRNA level following ESRRG knockdow **(C)** The RNA-seq heatmap shows the 168 DEGs in ESRRG-knockdown NOZ cells (n = 3). **(D)** Volcano plot of DEGs from RNA-seq showing 79 upregulated genes (red dots) and 89 downregulated (yellow dots) genes in ESRRG-knockdown NOZ cells (n = 3).The fold change is ≥2. **(E)** Top 20 signaling pathways enriched of DEGs following knockdown ESRRG based on KEGG enrichment analysis. **(F)** Top 30 signaling pathways enriched of DEGs following knockdown ESRRG based on GO enrichment analysis. *p < 0.05; **p < 0.01; p < 0.05 was considered statistically significant.

GSVA and PPI network analysis unveiled numerous genes potentially regulated by ESRRG. To elucidate these regulatory relationships, we examined the expression of these genes following ESRRG knockdown. As shown in **Figure 7A**, ESRRG knockdown led to decreased expression of MSH2 and BRCA1, implying that ESRRG may support DNA damage repair mechanisms, thereby influencing microsatellite instability and sensitivity to anti-PD-1/PD-L1 therapy. Elevated expression of TSC1 and TSC2 following ESRRG knockdown (**Figure 7A**) indicates enhanced inhibition of the mTOR signaling pathway. Furthermore, the expression of AKT1, RAF1, MAPK1, and MAPK14 decreased with ESRRG knockdown (**Figure 7A**), suggesting ESRRG’s role in promoting oncogenesis through the PI3K/AKT and MAPK pathways. Analysis of PPI network-associated genes revealed that ESRRG knockdown significantly decreased CREBZF and MED expression while boosting FGF9 and PNRC expression (**Figure 7B**), indicating that ESRRG may exert its biological effects through these genes. RNA-seq of ESRRG-knockdown NOZ cells identified 168 differentially expressed genes (DEGs; p < 0.05, |Log_2_FC| > 1), including 79 upregulated and 89 downregulated genes (**Figure 7D**). KEGG enrichment analysis showed significant enrichment of MAPK signaling and adherens junction pathways (**Figure 7E**). GO enrichment analysis highlighted immune system regulation, and biological adhesion (**Figure 7F**). These findings underscore ESRRG’s role in regulating pathways critical for GBC malignant progression and immune evasion.

## 3 Discussion

Pan-cancer analysis examines the functions of specific genes and gene families in all cancer types, providing researchers with a comprehensive perspective and valuable research insights. Pan-cancer analysis is an effective instrument for exploring the correlation of genes with immune regulation. It may investigate the impact of genes on immune function from various aspects, including immune-related genes, immunological checkpoints, cytokines, and immune-infiltrating cells. Due to the extensive use of second-generation sequencing technology, researchers have conducted a thorough exploration of the tumor microenvironment. Single-cell analyses have demonstrated that the composition of immune cells in the tumor immune microenvironment (TIME) alters, leading to immune escape (18). Precision immunotherapy based on TIME analysis has demonstrated positive outcomes in tumor treatment (19). Studying TIME is crucial for understanding the interactions between immune cells and cancer cells (20). The integration of machine learning and multi-omics data has also facilitated the discovery of new immune biomarkers and therapeutic targets. Machine learning algorithms based on immune-related genomics enable the prediction of immunotherapy sensitivity (21). Sahu et al. utilized in silico multi-omics approaches (BipotentR) to identify tumor-specific regulators of cancer immunity and discovered that ESRRA expression is significantly elevated in patients that are resistant to immunotherapy. Inhibiting ESRRA can suppress tumor proliferation by regulating metabolism and enhancing immune response (8). By mining multi-genomic analysis data, we comprehensively and systematically characterized ERRs in 19,131 samples across 44 cancers, and extensively analyzed the role of ERRs in tumors and immune-related mechanisms from a pan-cancer perspective.

Previous research has revealed that ERRs affect the progression of various cancers. These orphan nuclear receptors function by regulating mitochondrial function, target gene transcription, and hormone signaling. However, recent studies have underscored the significant effects of ERRs on immune regulation and the tumor microenvironment. For instance, inhibiting ESRRA enhances tumor cytotoxicity by promoting the recruitment of CD8^+^ T cells into the tumor microenvironment (8). In this study, we conducted a comprehensive pan-cancer analysis to elucidate the functions and mechanisms of the ERR family in various malignancies. Our findings reveal that ESRRA predominantly acts as an oncogene in multiple cancer types, whereas ESRRB and ESRRG exhibit distinct roles across different tumors. Comparative analysis with existing literature corroborates our results, indicating that ESRRA facilitates tumor progression in breast cancer (22, 23), pancreatic cancer (22), endometrial cancer (14) and GBC (9, 15). Conversely, ESRRB has been shown to inhibit breast cancer proliferation (11). In hepatocellular carcinoma, ESRRG functions as an oncogene, with its suppression leading to reduced cell proliferation via the induction of p21 and p27 (12). Additionally, antagonizing ESRRG triggers ferroptosis in sorafenib-resistant hepatocellular carcinoma (13). The ESRRG-PKM2 axis reprograms metabolism to exert a tumor-suppressive effect in esophageal squamous cell carcinoma (24). ESRRG also demonstrates tumor-inhibitory activity in gastric cancer (25). These findings validate the accuracy of our pan-cancer analysis.

Moreover, our study investigates the interplay between the ERR family and the tumor immune microenvironment, including tumor-infiltrating lymphocytes, immune checkpoints, and immune infiltration. The results indicate a significant correlation of ERRs with immune-related genes and cells. Specifically, in most cancer types, ESRRA positively correlates with TNFRSF14, and ESRRG with CD160. TNFRSF14 and CD160 function as an immune checkpoint stimulator and inhibitor (26), respectively, suggesting a close regulatory relationship between ERR genes and immune modulation. This implies that immune regulation may be one of the key mechanisms through which ESRRs exert their biological functions. MSI and TMB are crucial biomarkers for predicting the responsiveness of tumors to immune checkpoint inhibitors, such as anti-PD-1/PD-L1 therapies (27, 28). In this research, we found that ERRs are significantly associated with MSI and TMB across various cancer types, revealing the feasibility of modulating the activity of ESRRs to regulate the sensitivity of tumors to anti-PD-1/PD-L1 therapies.

Summarizing the results from our pan-cancer analysis, we have identified ESRRG as a pivotal factor in the progression of biliary tract tumors, including gallbladder cancer. Elevated ESRRG expression is linked to lymphatic metastasis, reduced overall survival, and decreased immune infiltration in CHOL. Specifically, in CHOL, ESRRG shows a negative association with TMB and a positive association with PD-L1 expression. Our pan-cancer analysis also reveals that the influence of ESRRG on immune cell infiltration is cancer-type specific. In THYM, ESRRG is associated with increased infiltration of immunosuppressive cells, such as M2 macrophages and regulatory T cells, facilitating tumor progression. Conversely, in LGG, ESRRG correlates with increased infiltration of NK cells and dendritic cells, thus promoting anti-tumor immunity.

In order to get more insight into the function of ESRRG in CHOL represented by gallbladder cancer, we performed validation studies on both cells and tissues. Our findings demonstrate that ESRRG knockdown in gallbladder cancer cells significantly impairs cell proliferation and migration, accompanied by a decrease in key elements of the EMT, MAPK, and mTOR signaling pathways. Moreover, knockdown of ESRRG led to decreased expression of MSH2 and BRCA1, suggesting that ESRRG might facilitate MSI, a critical marker for predicting the effectiveness of anti-PD-1/PD-L1 therapies. These results align with the GO enrichment analysis, which revealed significant enrichment of pathways associated with immune system processes. And these findings corroborates the discovery by Wang et al., demonstrating that ESRRG potentiates responsiveness to anti-PD-1 therapy in esophageal squamous cell carcinoma (24). Additionally, in GBC cells, PD-L1 expression was significantly reduced following ESRRG knockdown. This is the first study to report that ESRRG can positively regulate PD-L1 expression. The above results suggest that ESRRG positively regulates PD-L1 expression, thereby facilitating immune evasion and tumor progression. Increased PD-L1 expression can, in turn, enhance sensitivity to anti-PD-L1 therapies. Thus, the elevation of ESRRG in gallbladder cancer represents a double-edged sword: it promotes tumor progression while simultaneously enhancing responsiveness to anti-PD-1/PD-L1 therapies. Although our cellular and tissue experiments confirmed that ESRRG positively regulates PD-L1 expression, these findings have not been further validated through animal experiments. Future studies should prioritize not only optimizing the modulation of ESRRG but also investigating its impact on the sensitivity to anti-PD-L1 therapies using *in vivo* models. Additionally, it is noteworthy that gallbladder cancer exhibits marked sex disparities in incidence rates, with estrogen being an established risk factor. Given the homology between ERR family members (including ESRRG) and estrogen receptors, ESRRG may influence gallbladder cancer progression via involvement in estrogen signaling pathways, a hypothesis that merits further exploration.

Overall, ESRRG is a promising therapeutic target in gallbladder cancer and other biliary tract tumors. Targeting ESRRG could enhance immunotherapies by modulating the tumor immune microenvironment, reducing immune checkpoint expression, and restoring anti-tumor immunity. Further research is needed to elucidate ESRRG’s precise regulatory mechanisms and develop effective therapeutic strategies targeting ESRRG in cancer. In summary, we conducted a pan-cancer analysis of ERRs’ roles in tumors and explored their interactions with the tumor immune microenvironment, encompassing tumor-infiltrating lymphocytes, immune checkpoints, and immune infiltration. Furthermore, our study revealed that ESRRG promotes the progression of gallbladder cancer via inhibiting tumor immune microenvironment and, notably, for the first time, demonstrated a positive correlation between ESRRG expression and PD-L1 expression in gallbladder cancer.

## 4 Methods

### 4.1 Dataset and tumor types

We downloaded and incorporated the standardized pan-cancer dataset from the UCSC (https://xenabrowser.net/) database, including The Cancer Genome Atlas (TCGA), Therapeutically Applicable Research to Generate Effective Treatments (TARGET), and the Genotype-Tissue Expression (GTEx) (PANCAN, N=19131, G=60499), into our pan-cancer analysis. The dataset encompasses a variety of cancer types:TCGA-LAML and TARGET-LAML, ACC, BLCA, BRCA, CESC, CHOL, COAD, TCGA-COADREAD, ESCA, GBM, TCGA-GBMLGG, HNSC, KICH, KIRC, KIRP, KIPAN, LGG, LIHC, LUAD, LUSC, DLBC, MESO, OV, PAAD, PCPG, PRAD, READ, SARC, TCGA-SKCM, TCGA-SKCM-P, TCGA-SKCM-M, STAD, TCGA-STES, TGCT, THYM, THCA, UCS, UCEC, UVM, TARGET-WT, neuroblastoma (TARGET-NB) and TARGET-ALL, TARGET-ALL-R. Pan-cancer analysis was calculated in the platforms as follows: Sangerbox 3.0 (29) (http://vip.sangerbox.com/home.html), Xenashiny (https://shiny.hiplot.cn/ucsc-xena-shiny/), GSCALite (http://bioinfo.life.hust.edu.cn/web/GSCALite/), and GEMANIA (https://genemania.org/). From each sample, we retrieved the expression data of ERRs and subsequently screened the sample sources according to the specifications of each module. The expression value was converted using log2(x+0.001). Cancer types that did not have three or more samples were excluded from the analysis. Therefore, slight differences in the number of cancer types occurred across different analysis modules. For instance, the association analysis between ERR expression and immune regulation included 44 kinds of tumors, whereas the gene expression differential display comprised 34 kinds of tumors. For each analysis, we aimed to include as many cancer types as available in public databases to obtain more objective results.

### 4.2 ERR family gene expression differential display

The differential expressions of ESRRA, ESRRB, and ESRRG in 34 cancer types were analyzed in Sangebox 3.0 with the module: gene expression differential display. The sample sources were screened to include Solid Tissue Normal, Primary Solid Tumor, Primary Tumor, Normal Tissue, Primary Blood Derived Cancer-Bone Marrow, and Primary Blood Derived Cancer-Peripheral Blood samples. The R programming language, version 3.6.4, was used to compute the variations in expression between tumor and normal samples.

### 4.3 Exploring the correlation of the ERR family with pathological stages

The pan-cancer dataset was supplied by the TCGA database (PANCAN, N=10535, G=60499). The relationship between ERR expressions and clinical stages in 30 cancer types was analyzed using Sangebox 3.0. And the expression differences were computed applying R software (version 3.6.4).

### 4.4 Survival analysis and ROC curves

In Sangerbox 3.0, high-quality survival outcomes were derived from TCGA pan-cancer clinical data (30). Additionally, supplementary TARGET follow-up data was acquired from the UCSC (https://xenabrowser.net/datapages/). Cancer types with fewer than ten samples were removed, as were samples with a follow-up period of less than thirty days. Consequently, expression data for ERRs was obtained, corresponding to overall survival (OS) data of 44 kinds of tumors and progression-free survival (PFS) data of 38 kinds of tumors. To determine the association of ERRs with patient survival, we applied a Cox proportional hazards regression model. We performed univariate survival analysis with a Kaplan-Meier test among 32 cancer types of TCGA in Xena Shiny with the module TCGA: Survival Analysis. Next, the raw data of ERRs expression and prognosis from TCGA and GTEx in Sangerbox 3.0 were downloaded and imported into SPSS software to establish the ROC curve and evaluate the sensitivity and specificity of ESRRA, ESRRB, and ESRRG expression in predicting survival and progression.

### 4.5 Correlation analysis of ERR family genes and immune regulation

The correlation between ERR family genes and various immune signatures, including 24 immunoinhibitors, 46 immunostimulators, 18 chemokine receptors, 41 chemokines, and 21 MHC-related genes, was analyzed using Sangerbox 3.0. Data were collected from the following dataset: TCGA TARGET GTEx (PANCAN, N=19131, G=60499). Pearson correlation was calculated to assess the relationship between ERRs and representative genes from these immune pathways. Stromal, immune, and estimate scores were done by Sangerbox 3.0 (29). These metrics were derived from the analysis of 10,180 tumor specimens representing 44 kinds of cancers by utilizing the R package “ESTIMATE” (31). Spearman’s correlation coefficient was calculated using the corr.test function from the R package psych (version 2.1.6) between each gene in the ERR family and the immuneinfiltration score in each tumor. The purpose of this analysis was to identify significant associations of ERRs with immuneinfiltration scores.

### 4.6 Correlation analysis of ERR genes and immune cell infiltration

UCSC (https://xenabrowser.net/) provided pan-cancer datasets (TCGA TARGET GTEx (PANCAN, N=19131, G=60499)) regarding immune cell infiltration in 44 kinds of tumors for comparison. In Sangebox 3.0, the interrelationships between collated gene expression data and immune cell infiltration scores (encompassing neutrophils, T lymphocytes, macrophages, and lymphocytes) were evaluated by the “Timer” (32), “deconvo_CIBERSOR” (33), and “deconvo_QUANTISEQ” algorithms via the R package “IOBR” (34). Finally, we retrieved immunoinfiltration scores of 6 immune cells for the TIMER algorithm, 11 immune cells for the QUANTISEQ algorithm, and 22 immune cells for the CIBERSOR algorithm.

### 4.7 Interrelationship between ERRs and genomic heterogeneity

Tumor heterogeneity is vital for evaluating the sensitivity to immune checkpoint inhibitors (35). TMB, MSI, and cell stemness are the three main factors affecting tumor heterogeneity (36, 37). TCGA sample data of multiple tumors were downloaded for analysis. We used the modules that Association between molecular profile and TMB/Stemness/MSI in Xena Shiny (https://shiny.hiplot.com.cn/ucsc-xenashiny/) to evaluate the correlation of ERRs with TMB scores in 23 cancer types, MSI scores in 33 cancer types, and cell stemness scores in 33 cancer types. Spearman was performed to analyze the association of gene expression with TMB, MSI, and Stemness.

### 4.8 GeneMANIA analysis and pathway activity analysis

GeneMANIA helped to predict the functions of ERRs. PPI networks were further constructed (38, 39). GSCAlite pathway activity module calculated the correlation between ERR family genes and signaling pathways. Data sourced from The Cancer Proteome Atlas (TCPA) were utilized to derive scores for 7876 samples across 10 cancer-related pathways and 32 kinds of tumors, all originating from TCGA samples. The pathways under consideration encompassed TSC/mTOR, RTK, RAS/MAPK, PI3K/AKT, DNA Damage Response, Hormone ER, Hormone AR, EMT, Cell Cycle, and Apoptosis pathways. To ascertain the impact of genes on each pathway across the 32 cancer types, the percentage of cancers was computed as the ratio of the number of cancer types in which the gene either activated or inhibited the pathway to 32, multiplied by 100%.

### 4.9 Patients and specimens

This study included the collection of 50 gallbladder cancer tissue samples from Jiangnan University Medical Center between 2019 and 2023. The inclusion criteria for gallbladder cancer cases were: (a) confirmed pathological diagnosis, with staging according to the 8th edition of the AJCC staging system (40); (b) no neoadjuvant radiotherapy, chemotherapy, or immunotherapy administered prior to surgery; (c) radical resection of gallbladder cancer; (d) availability of complete clinical, pathological, and follow-up data. Additionally, 50 cholecystitis tissue samples were collected based on the following criteria: (a) confirmed pathological diagnosis; (b) undergoing laparoscopic cholecystectomy. The Jiangnan University Medical Center’s Ethics Committee approved this study (ID no. JNMC-EC-2023-Y-118), and each patient gave written, informed permission.

### 4.10 Immumohistochemical staining

Formalin-fixed, paraffin-embedded sections of gallbladder carcinoma and cholecystitis tissues were subjected to immunohistochemistry using a mouse anti-human ESRRG monoclonal antibody (OriGene, TA505080, Beijing, China). Briefly, the slides were baked in a thermostat at 65°C for 1.5 hours, then dewaxed and hydrated by xylene and different concentrations of ethanol, respectively. The antigens were repaired with sodium citrate repair solution (Solarbio, C1032, Beijing, China), and endogenous peroxidase was inactivated by blocking with 3% H_2_O_2_. 10% goat serum (Solarbio, SL038, Beijing, China) was used to block antigen for 30 minutes, and subsequently, the primary antibody against ESRRG (OriGene, TA505080, Beijing, China) was incubated at 4 °C overnight (41). After being incubated with the secondary antibody (DiagBio, db1003, Hangzhou, China), the sections were then stained with DAB (Solarbio, DA1015, Beijing, China) and counterstained with hematoxylin (Solarbio, G1140, Beijing, China).

On a scale of 0 to 3, the staining intensity was rated as follows: 0 (negative), 1 (weak), 2 (moderate), or 3 (strong). On a range of 0 to 4, the proportion of positive cells was evaluated as follows: 0 (negative), 1 (1-25%), 2 (26-50%), 3 (51-75%), or 4 (76-100%). Multiplying the intensity and percentage values resulted in the final immunereactivity score, which ranged from 0 to 12. Scores 0–3 were classified as “negative”, and 4–12 as “positive” (42).

### 4.11 Cell culture

Human gallbladder cancer EH-GB1 and OCUG cells were provided by Xinhua Hospital affiliated to Shanghai Jiaotong University School of Medicine. Human gallbladder cancer NOZ and GBC-SD cells were obtained from Cellverse Bioscience (Shanghai, China) and verified via STR profiling. These cells were grown in either DMEM (Hyclone, SH30022.01B, South Logan, UT, USA) or RPMI-1640 (Hyclone, SH30027.FS, South Logan, UT, USA) media, with additions of 10% fetal bovine serum (Gibco, A5669701, Grand Island, NY, USA), 100 μg/ml streptomycin, and 100 μg/ml penicillin (Hyclone, SV30010, South Logan, UT, USA). All cells were cultured in an incubator with 5% CO_2_ at 37°C.

### 4.12 RNA interference

Six-well plates were used to plant cells. Following the manufacturer’s recommendations, RFect reagent (BAIDAI, 11013, Changzhou, China) was applied to transfect shRNA as the cells in the culture plate had achieved 30–50% confluence. After 24 hours of transfection, RNA was extracted, and qPCR was used to verify the knockdown efficiency. Sangon Biotech (Shanghai, China) produced the sh-NC and sh-ESRRG sequences as follows: sh-ESRRG sense: CCUGUCAGGAAACUGUAUGAUTT; sh-ESRRG antisense: AUCAUACAGUUUCCUGACAGGT; sh-NC sense: GCGACGAUCUGCCUAAGAUTT; sh-NC antisense: AUCUUAGGCAGAUCGUCGCTT.

### 4.13 Western blot

Cellular proteins were isolated using a mixture of RIPA lysis buffer (Solarbio, R0010, Beijing, China) and PMSF (Solarbio, P0100-1, Beijing, China). After going through SDS-PAGE, proteins with the same concentration were put onto PVDF membranes. The resultant antibody-protein complexes were visible using enhanced chemiluminescence (ECL) reagents (Beyotime, P0018M, Nanjing, China) following incubation with primary and secondary antibodies. Protein quantification was performed using Image Lab and Image J software. The antibodies utilized included: ESRRG Mouse Monoclonal Antibody (OriGene, TA505080, Beijing, China); beta Actin (ACTB) Mouse Monoclonal Antibody (TA811000, OriGene, Beijing, China); LaminB1 Rabbit Polyclonal Antibody (OriGene, TA349381S, Beijing, China); PD-L1/CD274 Mouse Monoclonal antibody (Proteintech, No.66248-1-Ig, Wuhan, China); Goat Anti-Mouse IgG (H+L)-HRP (DiagBio, db1003, Hangzhou, China); and Goat Anti-Rabbit IgG (H+L)-HRP (DiagBio, db1002, Hangzhou, China).

### 4.14 Quantitative real-time PCR

Following the directions provided by the manufacturer, total RNA was extracted and purified using the RNA-easy Isolation Reagent (Vazyme, R701-02, Nanjing, China). Subsequently, the RNA was reverse-transcribed into cDNA with the HIScript III RT SuperMix (Vazyme, R323-01, Nanjing, China). Quantitative real-time PCR (qRT-PCR) was conducted on an ABI 7500 PCR system (Applied Biosystems, Foster City, CA, USA), utilizing Tap Pro Universal SYBR Master Mix (Vazyme, Q712-02, Nanjing, China). The PCR conditions included an initial denaturation at 95°C for 15 seconds, annealing temperature at 55-60°C for 15 seconds, extension at 72°C for 15 seconds, and total cycles of 45. 2^-ΔΔCt^ was applied to quantify the levels of gene expression, with GAPDH serving as the internal control. Primer sequences referred to were as follows: ESRRG-forward: CATGCTGAAAGAAGGGGTGC; ESRRG-reverse: CCACCAACAAATGTGAGACAATC; PD-L1-forward: TGCCGACTACAAGCGAATTACTG; PD-L1-reverse: CTGCTTGTCCAGATGACTTCGG; GAPDH-forward: AGAAGGCTGGGGCTCATTTG; GAPDH-reverse: AGGGGCATCCACAGTCTTC.

### 4.15 CCK-8 assay

The treated cells were plated at a density of 1000/well in 96-well plates with 100 μl medium containing 10% FBS. After 24h, 48h, 72h, 96h, and 120h, the old media was removed, respectively, and replaced with 100 μl fresh serum-free medium containing 10 μl CCK8 reagent (APExBIO, K2268-500T, Houston, USA). The cells were then cultured for an additional two hours in an incubator, and the OD value was measured at 450 nm. Each group included five replicates.

### 4.16 Colony formation assay

Cells from each experimental group were inoculated in 6-well plates with 1,000 cells/well, and then cultured in an incubator for approximately two weeks, with the culture medium replaced every three days. When most individual clonal clusters have more than 50 cells, the cells were fixed and stained using 4% paraformaldehyde (Solarbio, P1110, Beijing, China) and 0.1% crystal violet (Beyotime, C0121-100ml, Nanjing, China), respectively. Images were then captured, and Image J was used to handle the result.

### 4.17 Transwell assay

Trypsin-digested cells were resuspended in serum-free medium and counted, with the concentration being adjusted to 2×10^5/ml. In a 24-well plate with transwell inserts (Corning, 353097, NY, USA), 600 μl medium containing 20% FBS was added to the lower chamber, and 100 μl cell suspension was added to the upper chamber, which was placed in the incubator for further incubation. The cell invasion assay requires coating Matrigel (Corning, 356234, NY, USA) on the bottom inside the inserts in advance. 24 hours later, the inserts were taken out and fixed with 4% paraformaldehyde and stained with 0.1% crystal violet. Five arbitrary fields of view were captured with the microscope, and then the images were processed by Image J.

### 4.18 Wound healing assay

The transfected cells were grown into 6-well plates to achieve 90% fusion the next day. The cell surface was promptly scratched using pipette tips of 200 μl. After being washed twice with PBS, the cell was cultured in serum-free medium. The trauma was captured with the microscope at 0 and 24 h, respectively. The experimental results were analyzed using Image J.

### 4.19 Tissue processing and immunofluorescence staining

Samples of fresh tissue were preserved in 4% paraformaldehyde, embedded in paraffin under controlled temperature conditions, and sectioned onto slides. These slides were then roasted for two hours at 65°C. Pre-treatment involved sequential immersion in xylene, followed by washes in absolute ethanol, 85% ethanol, and 75% ethanol. Antigen retrieval was achieved using a high-temperature Tris-EDTA buffer (Beyotime, P0084, Nanjing, China). When these slides have cooled to ambient temperature, they were treated with a rapid protein-blocking solution (BOSTER, AR0041, Wuhan, China) for 1 hour. They were then incubated at room temperature for 2 hours with primary antibodies and, subsequently, for 1 hour with secondary antibodies conjugated with fluorophore. Finally, antifade mounting media with DAPI (Beyotime, P0131, Nanjing, China) was used to mount these sections. Imaging and analysis were performed using a confocal microscope (Nikon eclipse Ti2, Nikon, Tokyo, Japan) to capture detailed fluorescent signals. The main antibodies involved were as follows: PD-L1/CD274 Mouse Monoclonal antibody (Proteintech, 66248-1-Ig, Wuhan, China); CD8 Rabbit Monoclonal Antibody (BOSTER, BM4379, Wuhan, China); ESRRG Mouse Monoclonal Antibody (OriGene, TA504963, Beijing, China); Goat Anti-Rabbit IgG H&L (Alexa Fluor 594) (ZENBIO, 550043, Chengdu, China); Goat Anti-Mouse IgG H&L (Alexa Fluor^®^ 594) (Abcam, Ab150116, Cambridge, MA, USA); Goat Anti-Mouse IgG H&L (Alexa Fluor^®^ 488) preadsorbed (Abcam, Ab150117, Cambridge, MA, USA).

### 4.20 Transcriptomic analysis methods

Total RNA was extracted and purified using the RNA-easy Isolation Reagent (Vazyme, R701-02, Nanjing, China). RNA integrity was assessed using an Agilent 2100 Bioanalyzer (RIN > 7). Paired-end sequencing (PE150) was performed on an Illumina NovaSeq platform. Raw reads were quality-trimmed using fastp and aligned to the human reference genome (GRCh38) using STAR. Gene expression levels were quantified using feature Counts and analyzed for differential expression with DESeq2 (adjusted p-value < 0.05, |log_2_ fold change| > 1). Functional enrichment analysis was conducted using cluster Profiler for Gene Ontology (GO) and Kyoto Encyclopedia of Genes and Genomes (KEGG) pathways.

### 4.21 Statistical analysis

The differences in gene expression were processed through Unpaired Wilcoxon Rank Sum and Signed Rank Test. ANOVA was applied to conduct the difference test for multiple groups of samples. Correlations between two groups were calculated by the Spearman’s correlation test or the Pearson’s correlation test. The Cox proportional hazards model was employed for the multivariate survival analysis and the Kaplan-Meier for the univariate survival analysis. ROC curves for survival and progression were performed by SPSS 26.0. Statistical analysis. Quantitative data is shown as mean ± standard deviation (SD)/standard error (SE) for the experimental validation section. The means of the two groups were compared using an independent Student’s t-test. The χ² test assessed the correlation between ESRRG and clinicopathological parameters. A p-value of less than 0.05 was considered statistically significant. All experimental data were statistically analyzed through GraphPad Prism 8.3. All results are based on three independent replicates.

## Data Availability

The original contributions presented in the study are included in the article/**Supplementary Material**. Further inquiries can be directed to the corresponding authors.
