# Supplementary material for: Deciphering ERR family genes as prognostic and immunological biomarkers through pan-cancer analysis with validation in gallbladder cancer
Source: Front Oncol. 2025 Apr 28;15:1525635. doi: 10.3389/fonc.2025.1525635 (PMC12066295; doi:10.3389/fonc.2025.1525635)
Supplement: Supplementary file 1 [file DataSheet1.pdf]

Supplementary Material

1 Supplementary Figures and Tables

1.1 Supplementary Figures

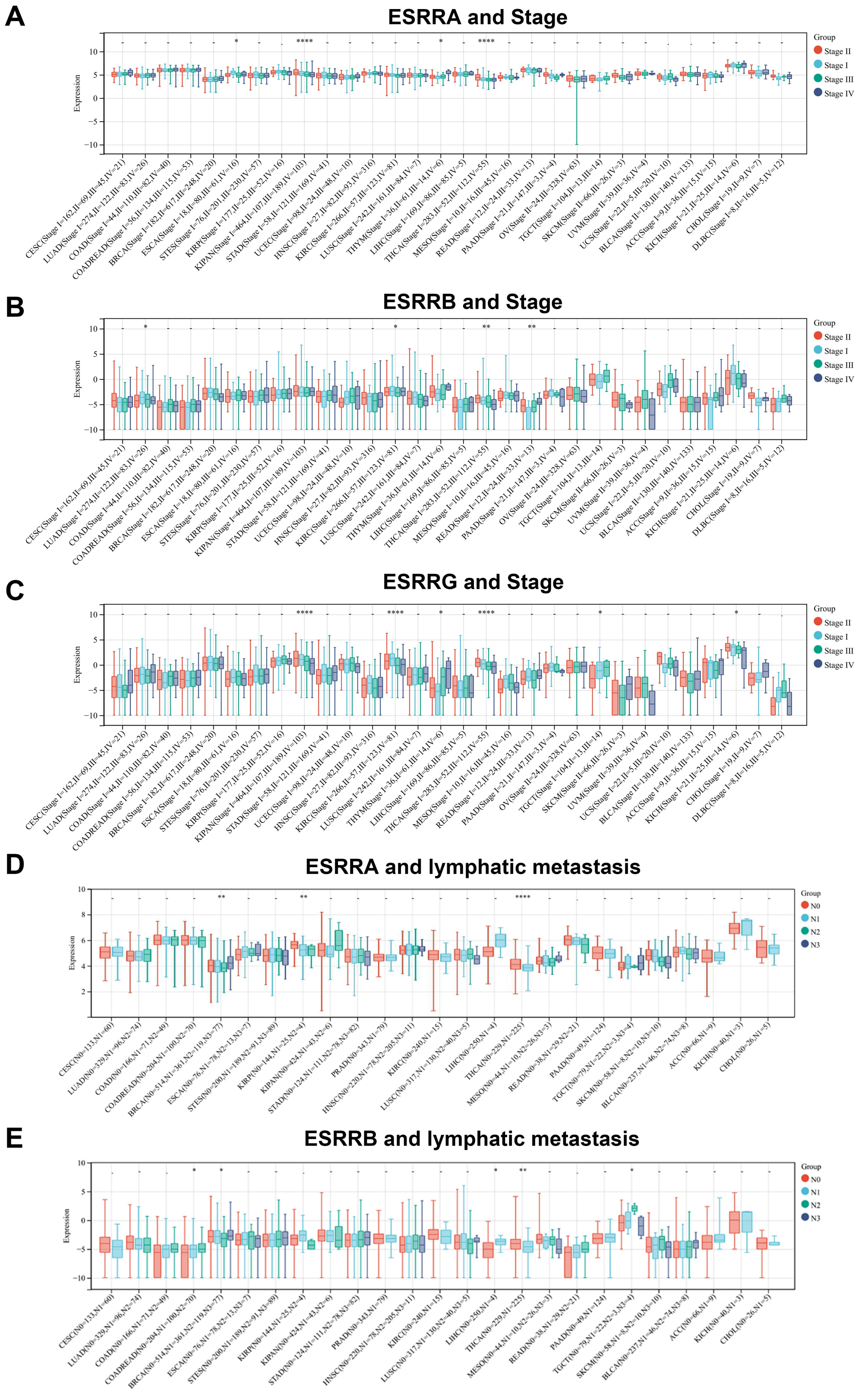
A
ESRRA and Stage
Group
Stage II
Stage I
Stage III
Stage IV
Expression
B
ESRRB and Stage
C
ESRRG and Stage
D
ESRRA and lymphatic metastasis
N0
N1
N2
N3
E
ESRRB and lymphatic metastasis

Supplementary Fig. S1. The correlation of ERRs with tumor stages and ymph node metastasis. (A-C) Expression of ESRRA (A), ESRRB (B), and ESRRG (C) at different pathological stages. (D-E) The association of ESRRA (A) and ESRRB (B) with lymph node metastasis in pan-cancer.

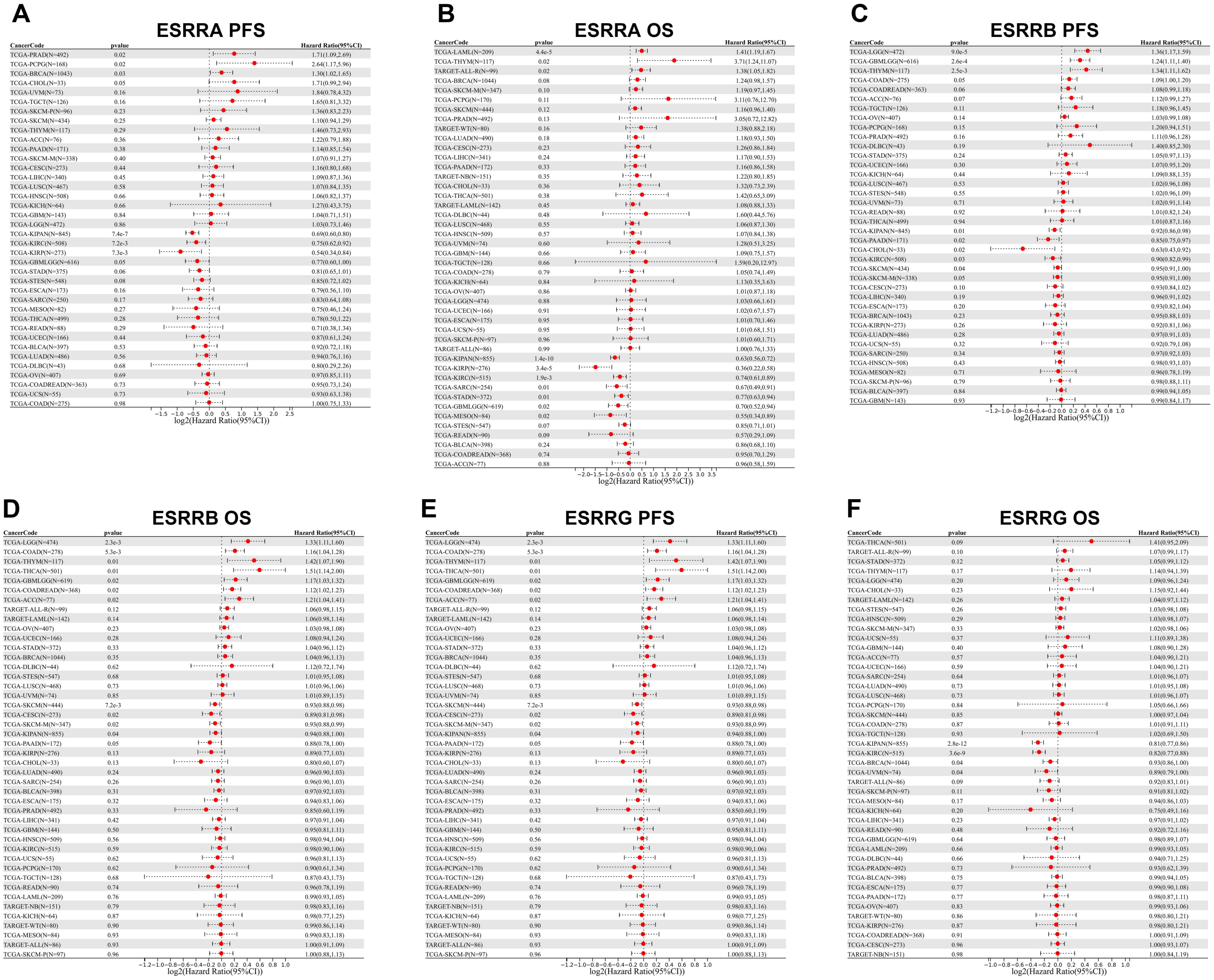

Supplementary Fig. S2: Associations between ERRs and PFS or OS were explored through COX regression analysis. (A-B) The association between ESRRA and tumor PFS (A) and OS (B). (C-D) The relationship between ESRRB and PFS (C) and OS (D). (E-F) The correlation between ESRRG and PFS (E) and OS (F).

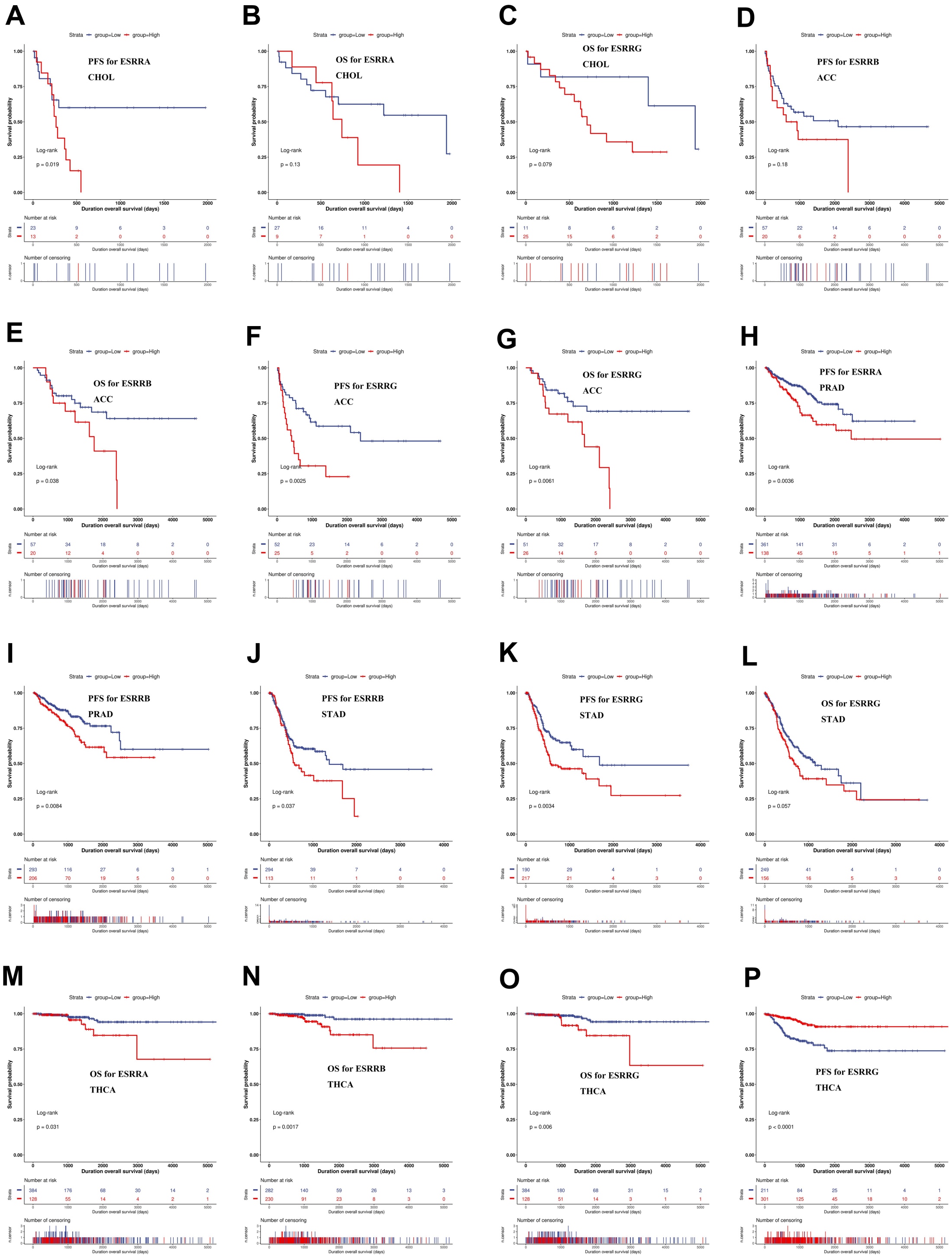

Supplementary Fig. S3: Kaplan-Meier survival analysis of ERRs with PFS and OS among cancers including CHOL (A-C), ACC (D-G), PRAD (H-I), STAD (J-L), and THCA (M-P).

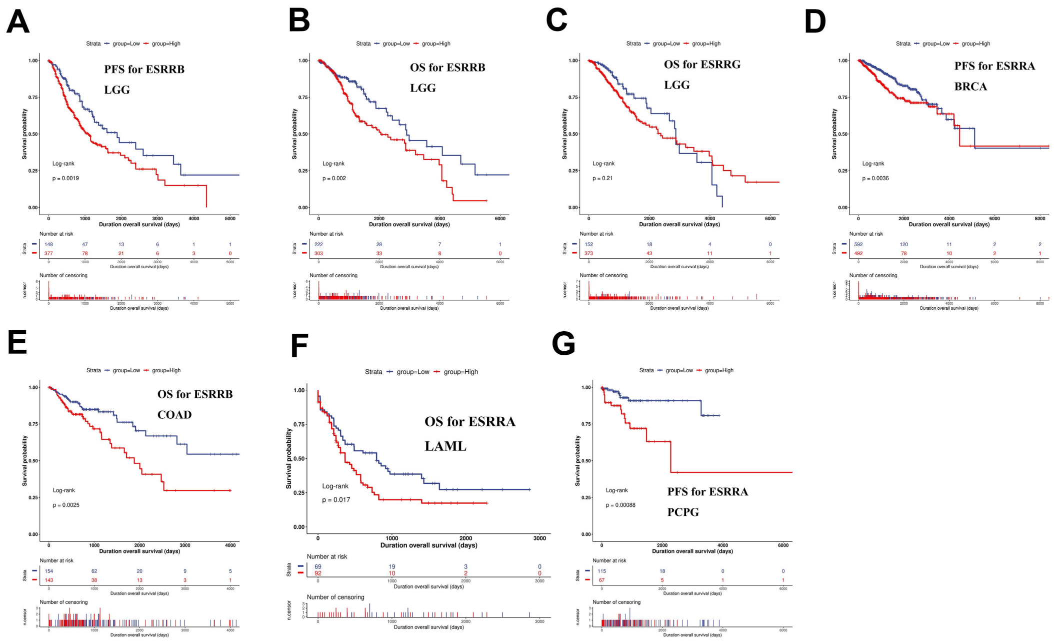

Supplementary Fig. S4. Kaplan-Meier survival analysis of ERRs with PFS and OS among cancers incliding LGG (A-C), BRCA (D), COAD (E), LAML (F), and PCPG (G).

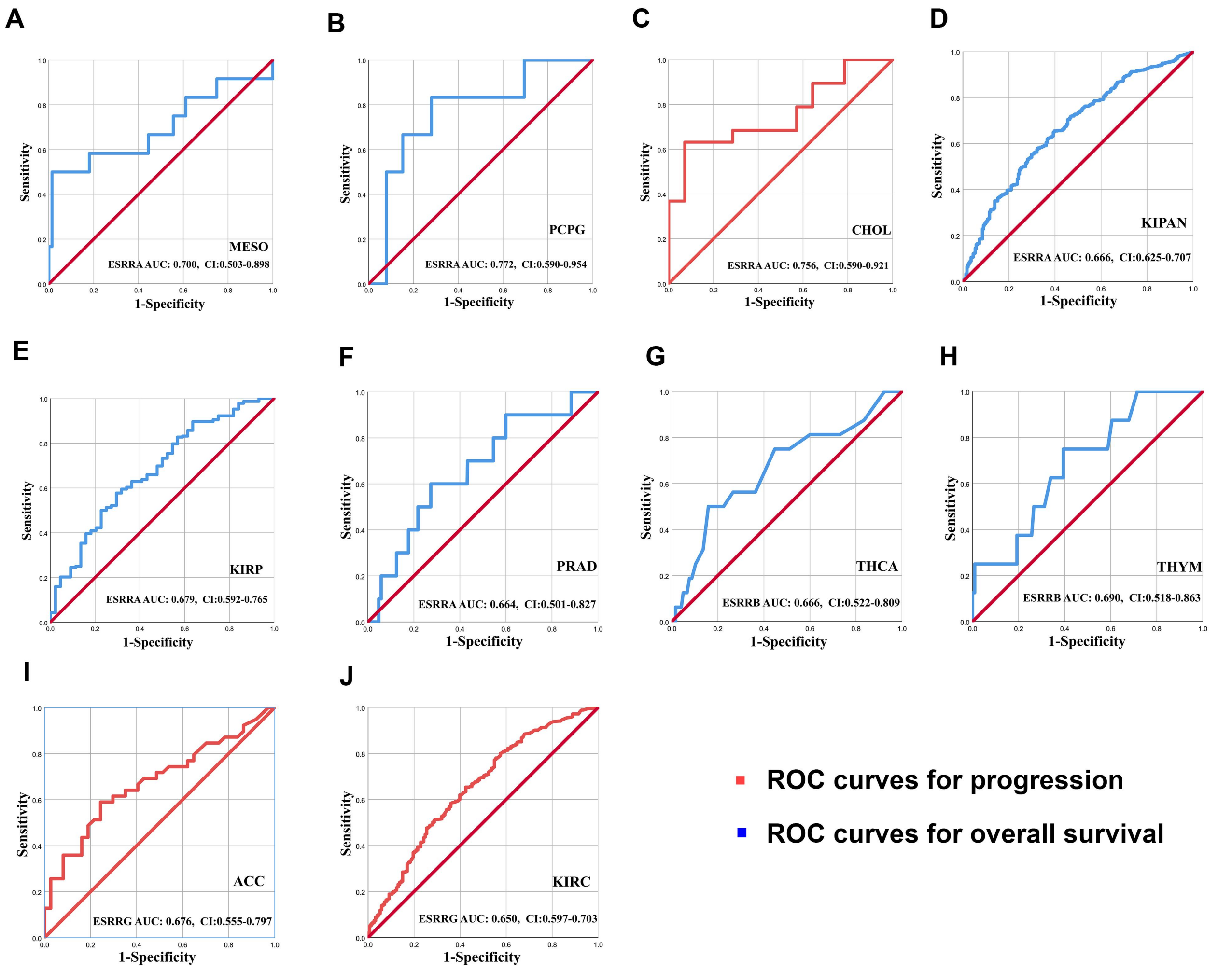

Supplementary Fig. S5.: ROC curves for ERRs. **(A-B, D-F)** Models with accuracy for survival predication were ESRRA for MESO (A), PCPG (B), KIPAN (D), KIRP (E) and PRAD (F). **(C)** Models with accuracy for the progression predication were ESRRA for CHOL. **(G-H)** Models with accuracy for survival predication were ESRRB for THCA (G) and THYM (H). **(I-J)** Models with accuracy for progression predication were ESRRG for ACC (I) and KIRC (J).

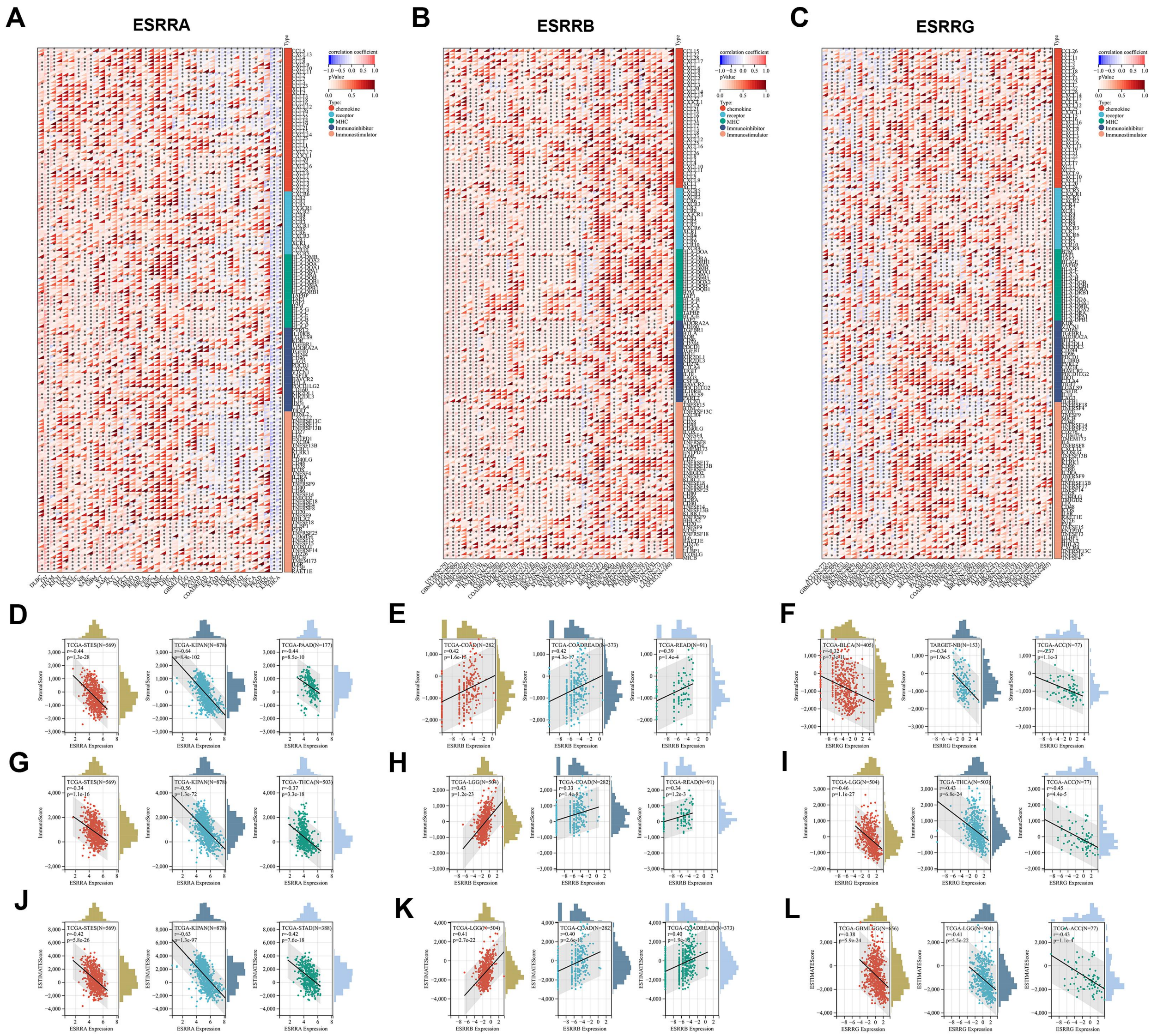

Supplementary Fig. S6: Relationship of ERRs with immune checkpoints and immune infiltration. **(A-C)** Correlation of ESRRA (A), ESRRB (B) and ESRRG (C) with immune checkpoint-related genes and immune infiltrating genes in pan-cancer. **(D-F)** The association of ESRRA (D), ESRRB (E) andESRRG (F) expression with Stromal Score. **(G-I)** The association of ESRRA (G), ESRRB (H) and ESRRG (I) expression with Immune Score. **(J-L)** The association of ESRRA (J), ESRRB (K) and ESRRG (L) expression with Estimate Score.

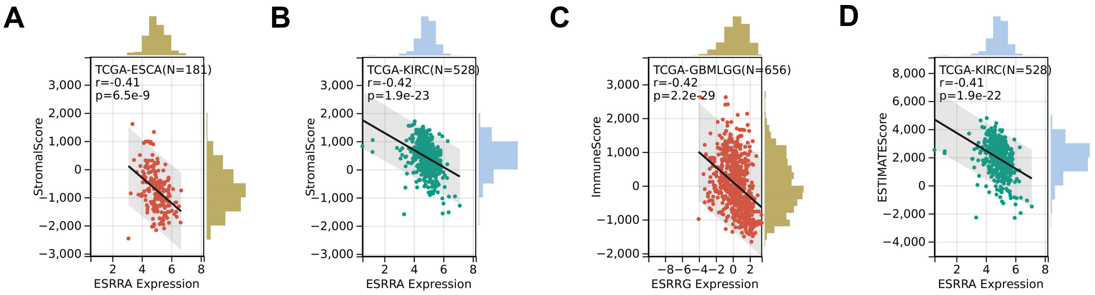

Supplementary Fig. S7: Relationship of ESRRA with immune infiltration. (A-B) The association of expression with Stromal Score in ESCA (A) and KIRC (B). (C) The association of ESRRA expression with Immune Score in GBMLGG. (D) The association of ESRRA expression with Estimate Score in KIRC.

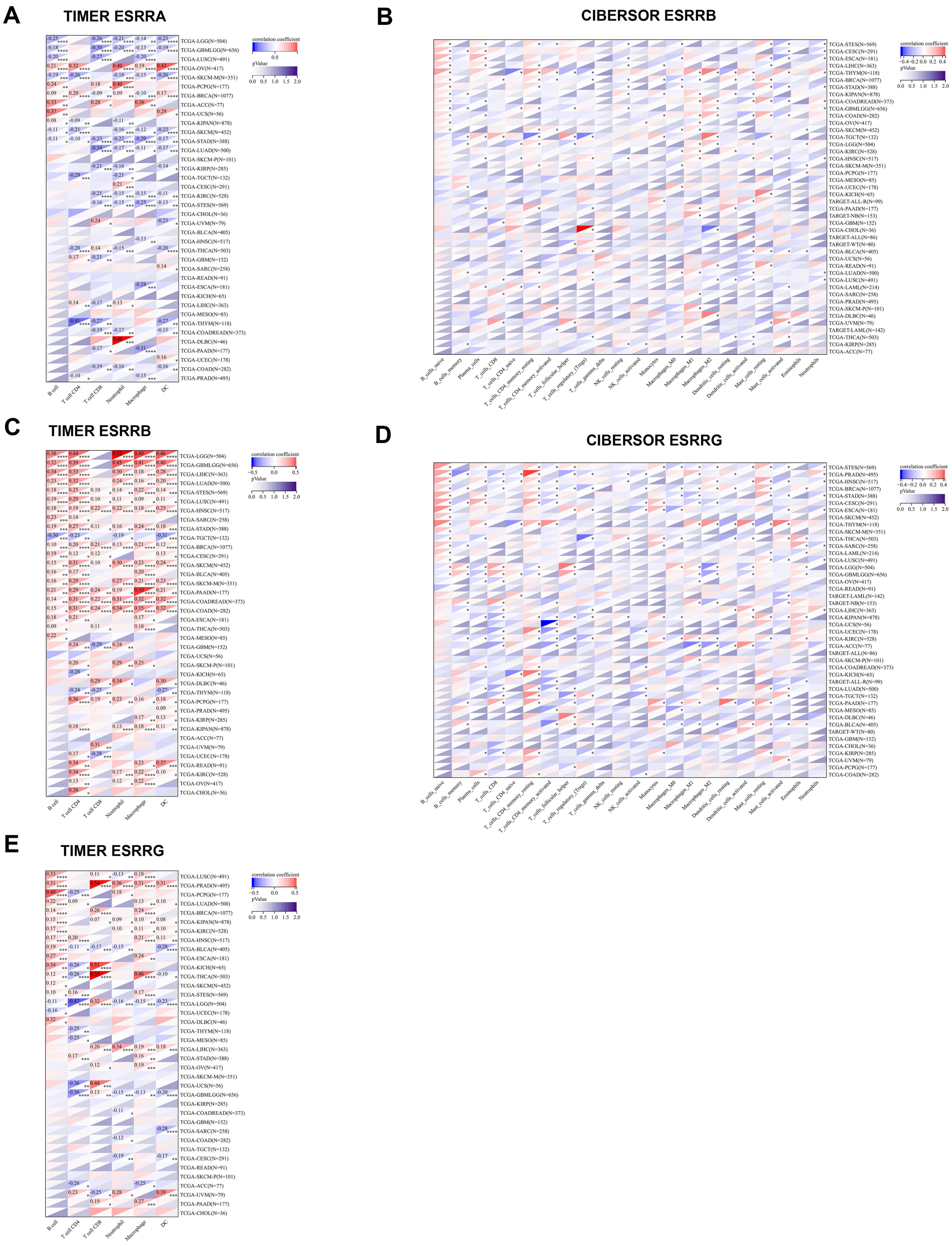

Supplementary Fig. S8. Correlation of ERRs with the level of immune infiltrating cells from pan-cancer. (A) Correlation of ESRRA with the level of immune infiltrating cells using TIMER algorithms. (B-C) Correlation of ESRRB with the level of immune infiltrating cells using CIBERSORT (B) and TIMER (C) algorithms. (D-E) Correlation of ESRRG with the level of immune infiltrating cells using using CIBERSORT (D) and TIMER (E) algorithms.

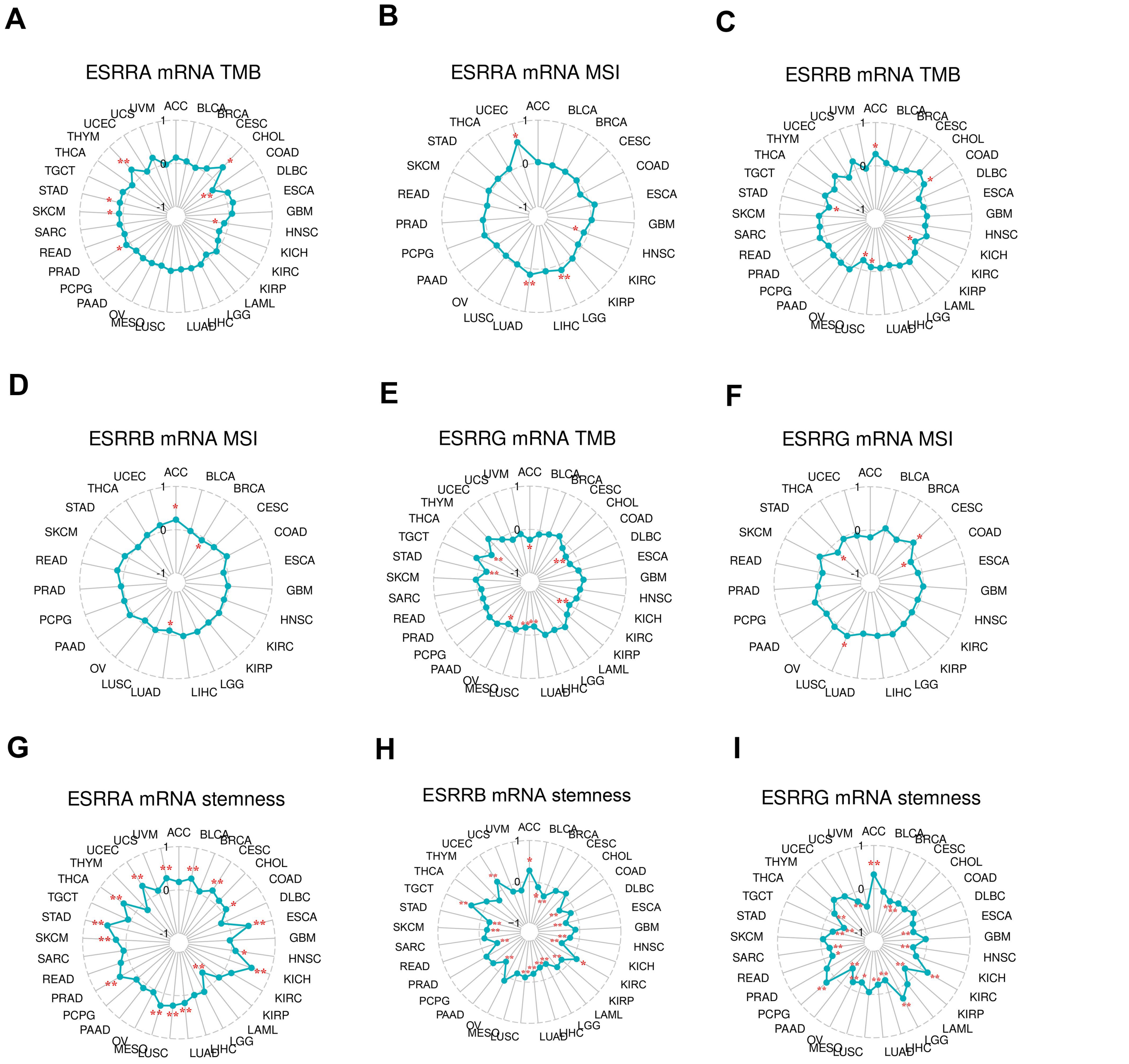

Supplementary Fig. S9: The correlation of ERRs mRNA expression with MSI, TMB, and stemness in pan-cancer. **(A-B)** The correlation of ERRA mRNA expression with TMB (A) and MSI (B). **(C-D)** The correlation of ERRB mRNA expression with TMB (C) and MSI (D). **(E-F)** The correlation of ERRG mRNA expression with TMB (E) and MSI (F). **(G-I)** The correlation between stemness scores and ESRRA (G), ESRRB (H) and ESRRG (I).

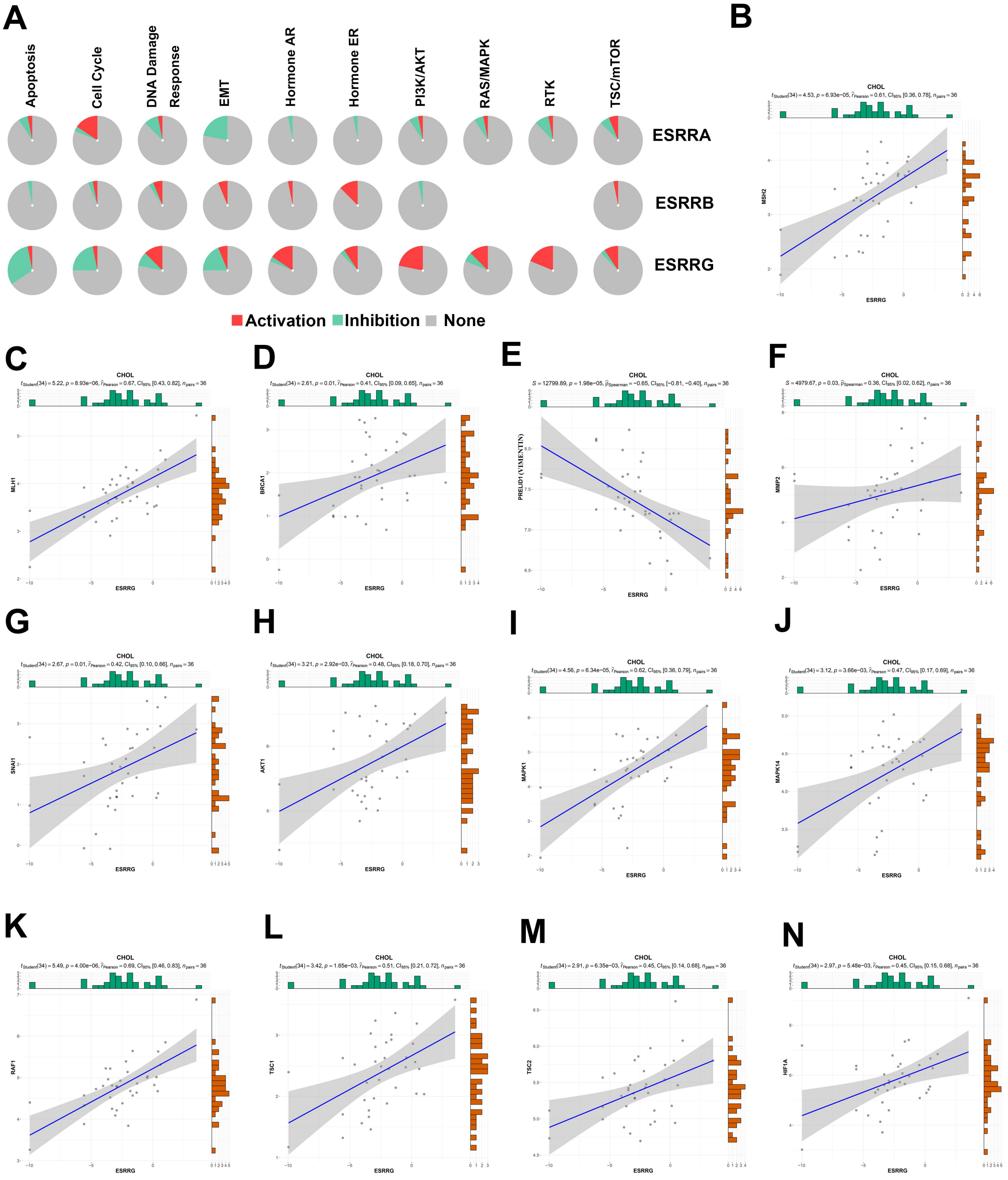

Supplementary Fig. S10: GeneMANIA analysis of ERRs-associated genes. **(A)** GSCAlite pathway activity module calculated the correlation between ERRs and signaling pathways. **(B-N)** Validation of the correlation between ESRRG expression and key regulators in the above signaling pathways in CHOL.

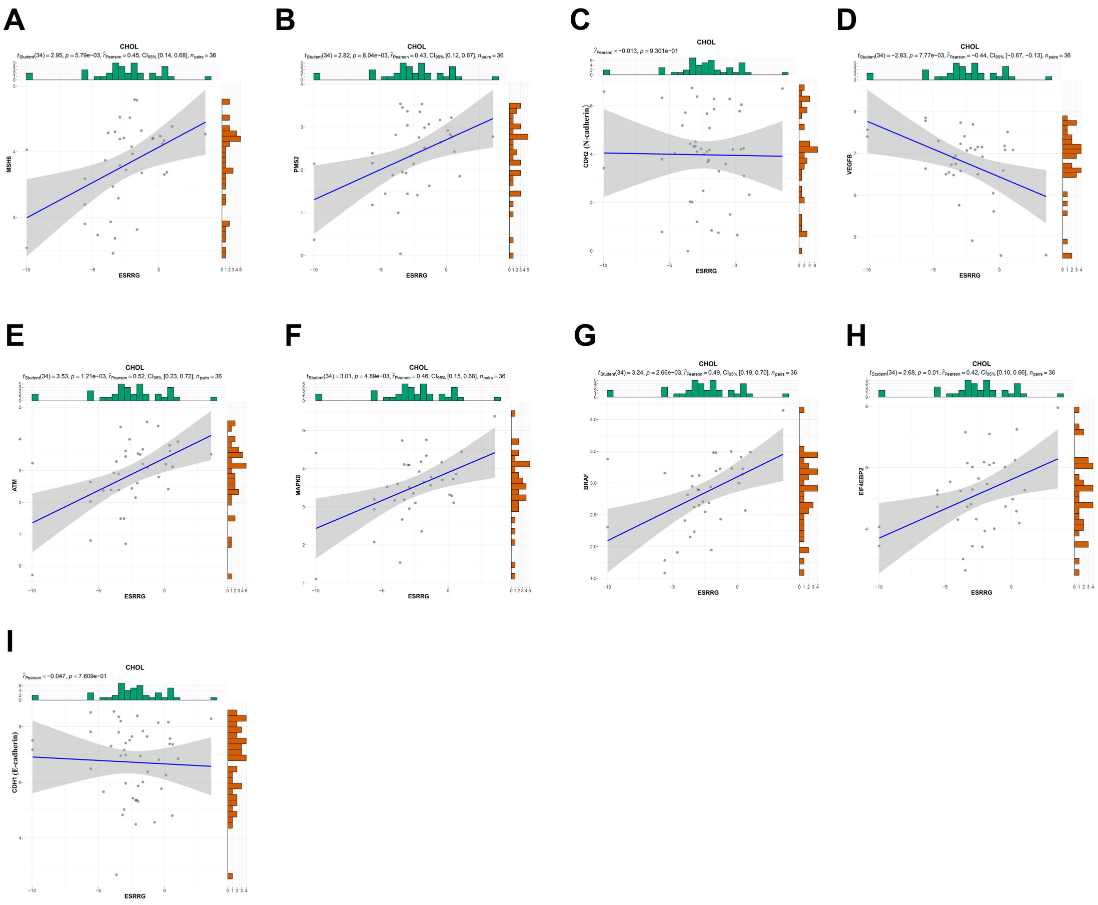

Supplementary Fig. S11: Validation of the correlation between ESRRG expression and key regulators of related signaling pathways from GSVA analysis in CHOL.

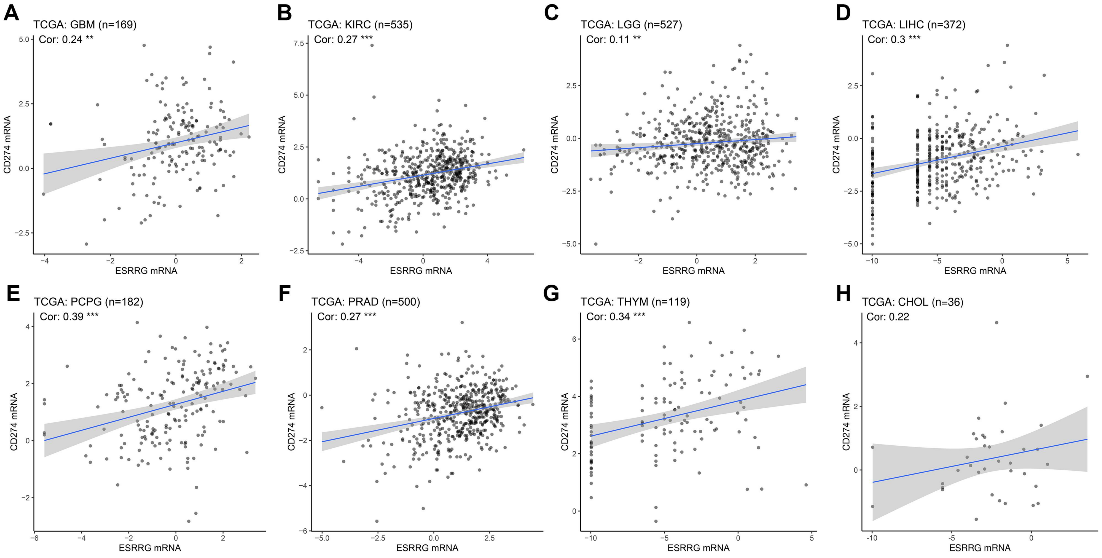

Supplementary Fig. S12: Positive correlations between the expression of PD-L1 and ESRRG in eight cancer types.

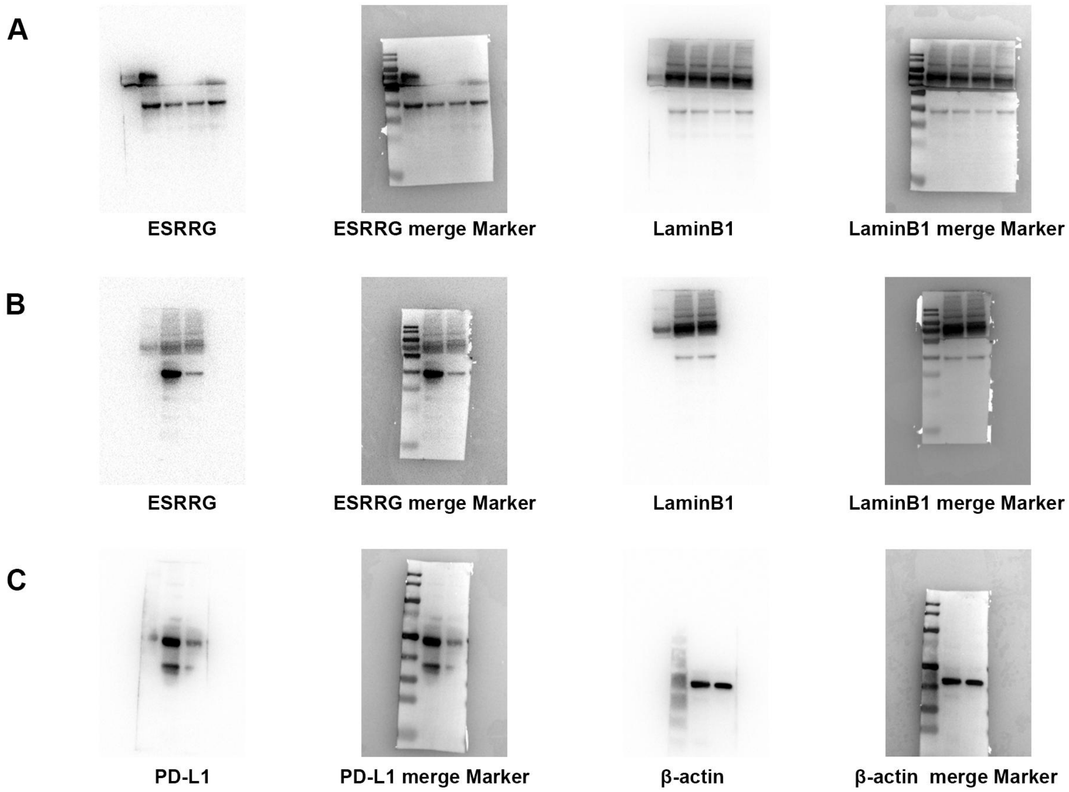

Supplementary Fig. S13: Representative original gels. (A) The original gels of Fig. 11B. The images in Fig. 11B were cropped from ESRRG and LaminB1. (B) The original gels of Fig. 11D. The images in Fig. 11D were cropped from ESRRG and LaminB1. (C) The original gels of Fig. 12B. The images in Fig. 12B were cropped from PD-L1 and β-actin.

1.2 Supplementary Tables

Supplementary Table 1. The raw data of R-value and P-value for ERRs' stromal score, immune score, and estimate score.

	ESRRA						ESRRB						ESRRG					
Immu	StromalScore	StromalScore	ImmuneScore	ImmuneScore	ESTIMATEScore	ESTIMATEScore	StromalScore	StromalScore	ImmuneScore	ImmuneScore	ESTIMATEScore	ESTIMATEScore	StromalScore	StromalScore	ImmuneScore	ImmuneScore	ESTIMATEScore	ESTIMATEScore
Method	spearman_R	spearman_P	spearman_R	spearman_P	spearman_R	spearman_P	spearman_R	spearman_P	spearman_R	spearman_P	spearman_R	spearman_P	spearman_R	spearman_P	spearman_R	spearman_P	spearman_R	spearman_P
TCGA-GBM(N=152)	-0.22	5.61E-03	-0.23	4.49E-03	-0.24	3.32E-03	0.00	9.60E-01	0.00	9.83E-01	-0.01	9.26E-01	-0.09	2.77E-01	-0.15	6.65E-02	-0.13	1.04E-01
TCGA-GBMLGG(N=656)	-0.26	8.38E-12	-0.21	7.36E-08	-0.23	1.92E-09	0.27	2.28E-12	0.31	1.42E-16	0.30	1.74E-15	-0.29	4.80E-14	-0.42	2.20E-29	-0.38	5.94E-24
TCGA-LGG(N=504)	-0.22	4.68E-07	-0.14	1.77E-03	-0.17	1.05E-04	0.37	8.61E-18	0.43	1.25E-23	0.41	2.69E-22	-0.29	1.62E-11	-0.46	1.05E-27	-0.41	5.54E-22
TCGA-UCEC(N=178)	-0.31	2.51E-05	-0.09	2.34E-01	-0.18	1.52E-02	-0.19	9.55E-03	-0.11	1.36E-01	-0.15	4.87E-02	-0.12	1.26E-01	-0.29	1.02E-04	-0.23	1.66E-03
TARGET-LAML(N=142)	0.08	3.30E-01	0.32	1.11E-04	0.23	5.76E-03	-0.27	1.06E-03	-0.13	1.35E-01	-0.19	2.23E-02	-0.22	8.76E-03	-0.09	2.64E-01	-0.16	6.55E-02
TCGA-BRCA(N=1077)	-0.10	7.85E-04	0.09	4.11E-03	0.00	9.35E-01	0.19	1.88E-10	0.05	1.12E-01	0.13	3.75E-05	0.04	2.04E-01	-0.16	9.40E-08	-0.09	3.93E-03
TCGA-CESC(N=291)	-0.21	2.39E-04	-0.04	5.25E-01	-0.13	2.61E-02	0.14	1.73E-02	0.04	4.99E-01	0.10	8.89E-02	-0.06	3.38E-01	-0.33	6.56E-09	-0.23	6.14E-05
TCGA-LUAD(N=500)	-0.35	1.97E-15	-0.28	9.76E-11	-0.35	1.18E-15	0.13	2.51E-03	0.18	5.84E-05	0.17	1.07E-04	-0.01	7.81E-01	-0.05	2.64E-01	-0.04	4.19E-01
TCGA-ESCA(N=181)	-0.41	6.51E-09	-0.18	1.58E-02	-0.32	9.99E-06	0.18	1.82E-02	0.14	5.48E-02	0.18	1.31E-02	0.06	4.35E-01	0.09	2.20E-01	0.08	2.61E-01
TCGA-STES(N=569)	-0.44	1.29E-28	-0.34	1.12E-16	-0.42	5.84E-26	0.15	2.67E-04	0.10	1.75E-02	0.14	1.12E-03	0.07	1.20E-01	0.03	4.32E-01	0.05	2.03E-01
TCGA-SARC(N=258)	0.05	3.95E-01	0.16	8.44E-03	0.12	6.49E-02	0.03	6.11E-01	0.06	3.58E-01	0.06	3.65E-01	-0.13	3.95E-02	-0.28	7.15E-06	-0.24	7.02E-05
TCGA-KIRP(N=285)	-0.22	1.49E-04	-0.19	9.76E-04	-0.21	2.79E-04	0.03	6.45E-01	-0.07	2.60E-01	-0.03	6.24E-01	-0.14	1.91E-02	-0.33	1.43E-08	-0.27	3.84E-06
TCGA-KIPAN(N=878)	-0.64	8.40E-102	-0.56	1.29E-72	-0.63	1.31E-97	0.08	1.64E-02	-0.01	8.81E-01	0.05	1.73E-01	-0.24	1.71E-12	-0.32	5.90E-23	-0.30	1.49E-19
TCGA-COAD(N=282)	-0.34	2.70E-09	-0.32	4.88E-08	-0.35	8.91E-10	0.42	1.60E-13	0.33	1.36E-08	0.40	2.62E-12	-0.01	8.29E-01	-0.08	1.69E-01	-0.05	4.11E-01

TCGA-COADREAD(N=373)	-0.31	6.38E-10	-0.29	7.08E-09	-0.32	1.56E-10	0.42	4.30E-17	0.33	7.26E-11	0.40	1.89E-15	0.00	9.93E-01	-0.08	1.09E-01	-0.04	4.27E-01
TCGA-PRAD(N=495)	-0.25	1.03E-08	-0.21	2.86E-06	-0.25	2.44E-08	-0.02	6.55E-01	-0.06	1.53E-01	-0.05	2.64E-01	-0.08	9.20E-02	-0.11	1.09E-02	-0.11	1.44E-02
TCGA-STAD(N=388)	-0.42	8.64E-18	-0.34	1.18E-11	-0.42	7.58E-18	0.17	8.46E-04	0.11	3.00E-02	0.15	3.13E-03	0.03	6.21E-01	-0.05	3.57E-01	-0.01	7.89E-01
TCGA-HNSC(N=517)	-0.21	1.11E-06	-0.16	3.92E-04	-0.21	2.45E-06	0.19	9.65E-06	0.18	2.70E-05	0.21	1.46E-06	0.16	1.67E-04	0.00	9.75E-01	0.08	7.50E-02
TCGA-KIRC(N=528)	-0.42	1.85E-23	-0.32	2.80E-14	-0.41	1.93E-22	0.12	6.63E-03	0.05	2.40E-01	0.10	2.13E-02	-0.15	3.59E-04	-0.21	1.12E-06	-0.21	7.21E-07
TCGA-LUSC(N=491)	-0.32	1.74E-13	-0.22	6.72E-07	-0.29	5.43E-11	0.07	1.47E-01	0.10	2.42E-02	0.09	4.04E-02	-0.01	8.32E-01	-0.06	1.88E-01	-0.04	3.92E-01
TCGA-THYM(N=118)	-0.12	1.84E-01	-0.24	8.76E-03	-0.28	2.49E-03	0.17	7.16E-02	0.03	7.21E-01	0.08	4.01E-01	-0.07	4.33E-01	0.04	6.32E-01	-0.07	4.31E-01
TCGA-LIHC(N=363)	-0.08	1.06E-01	-0.23	1.18E-05	-0.18	4.13E-04	0.23	1.48E-05	0.09	7.17E-02	0.16	1.74E-03	0.12	1.88E-02	0.02	7.11E-01	0.07	1.86E-01
TARGET-WT(N=80)	-0.14	2.23E-01	-0.25	2.57E-02	-0.23	3.63E-02	0.10	3.97E-01	-0.07	5.16E-01	-0.01	9.55E-01	0.09	4.05E-01	-0.35	1.57E-03	-0.14	2.17E-01
TCGA-SKCM-P(N=101)	-0.26	9.16E-03	-0.21	3.39E-02	-0.24	1.53E-02	0.03	7.38E-01	0.13	2.04E-01	0.12	2.47E-01	-0.13	1.81E-01	-0.19	5.11E-02	-0.18	7.18E-02
TCGA-SKCM(N=452)	-0.31	1.56E-11	-0.29	2.38E-10	-0.32	2.95E-12	0.20	2.76E-05	0.17	3.05E-04	0.20	2.55E-05	-0.04	4.15E-01	-0.13	7.75E-03	-0.10	3.99E-02
TCGA-BLCA(N=405)	-0.33	9.38E-12	-0.18	3.71E-04	-0.28	1.48E-08	0.08	1.12E-01	0.06	2.37E-01	0.07	1.35E-01	-0.32	7.35E-11	-0.31	2.31E-10	-0.34	4.15E-12
TCGA-SKCM-M(N=351)	-0.32	6.36E-10	-0.31	1.62E-09	-0.34	4.39E-11	0.22	3.51E-05	0.16	3.13E-03	0.20	1.96E-04	-0.05	3.37E-01	-0.15	5.32E-03	-0.12	2.57E-02
TCGA-THCA(N=503)	-0.34	5.32E-15	-0.37	3.32E-18	-0.39	1.57E-19	0.02	5.88E-01	-0.05	2.99E-01	-0.02	6.80E-01	-0.20	5.70E-06	-0.43	6.82E-24	-0.35	4.55E-16
TARGET-NB(N=153)	0.10	2.16E-01	0.20	1.20E-02	0.17	3.48E-02	0.22	6.14E-03	0.31	1.04E-04	0.30	1.50E-04	-0.34	1.89E-05	-0.14	7.70E-02	-0.25	1.76E-03
TCGA-MESO(N=85)	-0.19	7.39E-02	0.02	8.59E-01	-0.09	4.23E-01	0.06	6.10E-01	-0.11	3.12E-01	-0.07	4.98E-01	-0.19	7.76E-02	-0.22	4.77E-02	-0.23	3.19E-02
TCGA-READ(N=91)	-0.18	9.00E-02	-0.22	3.59E-02	-0.22	3.67E-02	0.39	1.38E-04	0.34	1.16E-03	0.38	2.21E-04	0.03	7.43E-01	-0.10	3.41E-01	-0.02	8.20E-01
TCGA-OV(N=417)	0.06	2.29E-01	0.20	3.01E-05	0.14	3.67E-03	-0.10	3.50E-02	-0.19	8.71E-05	-0.16	1.42E-03	-0.11	2.70E-02	-0.20	2.99E-05	-0.17	5.81E-04
TCGA-UVM(N=79)	-0.25	2.64E-02	-0.14	2.34E-01	-0.19	1.01E-01	0.22	5.09E-02	0.25	2.92E-02	0.25	2.65E-02	-0.02	8.31E-01	-0.13	2.72E-01	-0.10	3.70E-01

TCGA-PAAD(N=177)	-0.44	8.52E-10	-0.30	4.37E-05	-0.40	3.87E-08	0.24	1.42E-03	0.17	2.56E-02	0.21	5.66E-03	0.13	7.71E-02	0.10	1.90E-01	0.11	1.44E-01
TCGA-TGCT(N=132)	-0.01	9.46E-01	-0.24	5.31E-03	-0.17	5.00E-02	-0.01	8.82E-01	-0.26	2.69E-03	-0.20	2.01E-02	0.08	3.37E-01	-0.11	2.15E-01	-0.05	5.82E-01
TCGA-UCS(N=56)	0.18	1.73E-01	0.12	3.82E-01	0.19	1.69E-01	0.03	8.22E-01	-0.26	4.99E-02	-0.14	2.90E-01	0.06	6.53E-01	-0.29	2.86E-02	-0.17	2.17E-01
TCGA-LAML(N=214)	0.49	1.95E-14	0.27	5.88E-05	0.38	7.24E-09	0.00	9.45E-01	0.08	2.49E-01	0.05	4.45E-01	-0.02	7.26E-01	0.07	2.98E-01	0.04	5.43E-01
TARGET-ALL(N=86)	-0.10	3.83E-01	-0.10	3.73E-01	-0.13	2.25E-01	-0.13	2.41E-01	-0.35	8.04E-04	-0.30	5.69E-03	0.22	4.57E-02	0.05	6.48E-01	0.17	1.19E-01
TCGA-PCPG(N=177)	-0.03	6.64E-01	0.00	9.73E-01	-0.03	7.41E-01	0.19	1.31E-02	0.08	2.77E-01	0.14	7.23E-02	-0.11	1.59E-01	0.04	6.28E-01	-0.04	6.43E-01
TCGA-ACC(N=77)	-0.13	2.77E-01	-0.03	7.78E-01	-0.06	6.02E-01	-0.21	6.22E-02	-0.13	2.46E-01	-0.17	1.37E-01	-0.37	1.06E-03	-0.45	4.44E-05	-0.43	1.06E-04
TARGET-ALL-R(N=99)	0.33	9.14E-04	0.27	7.88E-03	0.30	2.45E-03	0.10	3.24E-01	0.16	1.08E-01	0.14	1.57E-01	-0.26	1.01E-02	-0.22	3.11E-02	-0.24	1.92E-02
TCGA-DLBC(N=46)	0.15	3.26E-01	0.18	2.22E-01	0.19	2.06E-01	0.22	1.50E-01	0.34	2.09E-02	0.39	7.29E-03	0.01	9.54E-01	0.15	3.26E-01	0.10	5.20E-01
TCGA-KICH(N=65)	-0.01	9.15E-01	0.12	3.33E-01	0.07	5.53E-01	-0.18	1.49E-01	-0.24	5.82E-02	-0.22	7.42E-02	-0.18	1.48E-01	-0.28	2.44E-02	-0.25	4.43E-02
TCGA-CHOL(N=36)	-0.25	1.41E-01	-0.23	1.70E-01	-0.26	1.32E-01	0.07	6.80E-01	0.04	8.33E-01	0.05	7.67E-01	0.03	8.59E-01	-0.16	3.51E-01	-0.08	6.42E-01
